# Genome-wide systematic characterization of *bZIP* transcription factors and their expression profiles during stem in tumorous stem mustard

**DOI:** 10.7717/peerj.20518

**Published:** 2026-01-14

**Authors:** Yuting Deng, Fu Li, Yongfang Xie, Jiaxin Guo, Jianzhong Shu, Rong Qin, Quan Sun, Keman Wu, Feibo Xu, Xiaohong He

**Affiliations:** 1 Chongqing Key Laboratory of Big Data for Bio Intelligence, School of Life Health Information Science and Engineering, Chongqing University of Post and Telecommunications, Chongqing, China; 2 Chongqing Hospital of Traditional Chinese Medicine, Chongqing, China

**Keywords:** *Brassica juncea var. Tumida*, bZIP gene family, Transcription factors, Bioinformatics

## Abstract

The Basic Leucine Zipper (bZIP) proteins constitute a large family of transcription factors that play critical roles in plant growth regulation and the expression of resistance genes. However, to date, there have been few reports on the *bZIP* family in tumorous stem mustard (*Brassica juncea var. tumida*), an important vegetable crop. In this study, we identified 153 *bZIP* genes in tumorous stem mustard, which are unevenly distributed across 18 chromosomes and form 13 gene clusters. We systematically investigated their protein characteristics, phylogenetic relationships, gene structures, and conserved motifs. Most bZIP proteins exhibited random coil and α-helix as their predominant secondary structures. Based on RNA-Seq data from our laboratory, we analyzed the expression profiles of *bZIP* genes during the stem expansion of tumorous stem mustard. Furthermore, qRT-PCR analysis was performed to validate the expression of selected *bZIP* genes in tumorous stem mustard. The results showed that, compared with the 0 h control (25 °C untreated seedlings), five *BjubZIP* genes were significantly upregulated and eight were downregulated after 48 h of cold treatment, suggesting their potential involvement in temperature stress regulation in tumorous stem mustard. Under heat-treatment conditions, the transcription levels of *BjubZIP111* and *BjubZIP070* increased continuously over time, with expression at 48 hours significantly higher than at 12 hours. These findings provide a foundation for further functional research on *bZIP* genes in tumorous stem mustard, as well as for its breeding and production. Additionally, this study offers a theoretical basis for functional genomics research and the development of new cultivars of tumorous stem mustard.

## Introduction

Tumorous stem mustard (*Brassica juncea var. tumida*, 2n = 36, AABB) is the offspring of cabbage (*Brassica rapa*, 2n = 20, AA) and black mustard (*Brassica nigra*, 2n = 16, BB) formed by double heteropolyploidy after natural hybridization (Yang et al., 2018). In the long-term evolution process, the vegetable-use and oil-use subvarieties mainly evolved into two categories (Yang et al., 2016), the former was mainly distributed in China and other East Asian countries and regions, and the latter was mainly distributed in India and other South Asian countries and regions (Kang et al., 2021). In China, the tumorous stem mustard in vegetable mustard is largely planted in Sichuan and Zhejiang provinces. The soil and climate conditions in Fuling, Chongqing are most suitable for the growth of tuberous mustard. The tuberous stem formed is particularly fertile and tender. It is the main raw material for making Fuling mustard and has high edible value and development value.

Transcription factor (TF) is a kind of protein that can bind to specific DNA sequences to control the rate of transcription of DNA genetic information to messenger RNA (Latchman, 1997). The function of transcription factors is to regulate gene expression and to ensure that all genes with the right amount are expressed at the correct time in the whole life cycle of organisms (Karin, 1990). Transcription factors (TFs) have a momentous impact on the regulation of various life activities. The Basic Leucine Zipper (bZIP) transcription factor is one of the largest and most conservative gene families in plant transcription factors, composed of 60∼80 conservative amino acid residues, with the typical dimer structure characteristics (Nijhawan et al., 2008). The C-terminal of the bZIP proteins possesses a basic amino acid, while the N-terminal possesses a leucine zipper, both of them are conservative bZIP domain structures (Talanian, McKnight & Kim, 1990). The basic amino acid region contains a region for the localization of the nucleus and a domain for DNA recognition immediately thereafter. The leucine zipper region is composed of two typical alpha helices, and every alpha helix has one leucine or another hydrophobic amino acid and every 6 amino acid residues. Leucine with a strongly hydrophobic group can often dimerize these two alpha helices before the bZIP protein binds to DNA, forming a homodimer or heterodimer (Ellenberger et al., 1992; Landschulz, Johnson & McKnight, 1988).

Numerous studies have revealed that plant *bZIP* transcription factors play important regulatory functions in life processes such as seed development (Izawa et al., 1994; Toh, McCourt & Tsuchiya, 2012), flower development (Abe et al., 2005; Gibalova et al., 2009; Iven et al., 2010), carbon and nitrogen energy metabolism, and biological and abiotic stress responses (Wang et al., 2011). In particular, plants grown in adversity depend on the regulation of *bZIP* transcription factors, showing strong resistance to disease, salt, and drought. Under stress conditions, the *bZIP* transcription factor participates in abscisic acid (ABA) response and interacts with corresponding Abscisic Acid-Responsive Element (ABRE) binding factors (ABFs) or ABRE binding proteins to regulate the transcriptional expression of downstream target genes (Fujita et al., 2005; Guiltinan, Marcotte Jr & Quatrano, 1990). At present, the *bZIP* transcription factor family has been identified in various plants. For example, the model organism *Arabidopsis thaliana* holds 75 *bZIP* transcription factors (Jakoby et al., 2002), which have also been found in the genomes of rice, corn, sorghum, soybean, and tomato, respectively, 89 (Nijhawan et al., 2008), 125 (Wei et al., 2012), 92 (Wang et al., 2011), 131 (Liao et al., 2008) and 69 (Li et al., 2015) *bZIP* transcription factors.

As a kind of vegetable, tumorous stem mustard plays a decisive role in the production of mustard. However, even to this day, the genome-wide characteristics of *bZIP* transcription factors in stem mustard have not been systematically studied. To comprehensively analyze the characteristics and biological functions of the *bZIP* transcription factor family in tumorous stem mustard, this study identified *bZIP* sequences at the whole-genome level, obtaining 153 *BjubZIP* transcription factor proteins. Using bioinformatics tools, we predicted and analyzed phylogenetic relationships, protein physicochemical properties, conserved amino acid domains, gene duplications, and other features. Additionally, transcriptome sequencing data from this study were used to examine expression patterns during the stem expansion of tumorous stem mustard. These findings provide a theoretical basis for functional research and contribute to the breeding of tumorous stem mustard.

## Materials & Methods

### Data acquisition and material preparation

The genome sequence, protein sequence, and genome annotation files of tumorous stem mustard (*Brassica juncea L.*) were all obstained from the Brassicaceae Database (http://brassicadb.cn/, BRAD) (Chen et al., 2022). The *At*bZIP protein sequences of *Arabidopsis thaliana* were downloaded from The Arabidopsis Information Resource (https://www.arabidopsis.org/, TAIR) (Berardini et al., 2015). The tumorous stem mustard cultivar ‘Yongan leaflets’ was selected and grown in the Molecular Biology Laboratory of Chongqing University of Posts and Telecommunications.

### Genome identification

Three hidden Markov model (HMM) profiles corresponding to *bZIP* transcription factor domains (bZIP_1: PF00170, bZIP_2: PF07716, bZIP_Maf: PF03131) were retrieved from the Pfam database (http://pfam.xfam.org/) (Mistry et al., 2021). HMMER 3.0 software (http://hmmer.org/) (Finn, Clements & Eddy, 2011) was used to search for bZIP proteins in the tumorous stem mustard protein sequence files, with the *E*-value set to e-10 and default parameters applied. The ClustalW multi-sequence alignment result file was used to construct a bZIP structural domain hidden Markov model specific to tumorous stem mustard. A secondary search was then performed on the aforementioned sequences, which were screened based on an *E*-value threshold of less than 0.001, with repetitive sequences removed. All protein sequences obtained from the HMMER results, corresponding to gene IDs, were uploaded to the NCBI Conserved Domain Database (CDD) (https://www.ncbi.nlm.nih.gov/cdd/), the Pfam database, and the SMART database (http://smart.embl.de/) (Letunic, Khedkar & Bork, 2021) for further identification. This step was conducted to confirm the presence or absence of the conserved bZIP domain in candidate protein sequences, eliminate candidate genes lacking the bZIP domain, and ultimately obtain the *bZIP* transcription factor protein sequences of tumorous stem mustard. The identified bZIP protein sequences of tumorous stem mustard were submitted to the Bologna Unified Subcellular Component Annotator (BUSCA) (http://busca.biocomp.unibo.it/) (Savojardo et al., 2018) for the subcellular localization prediction.

### Phylogenetic analysis and classification of tumorous stem mustard *bZIP* gene family

Extract the conserved domain sequences of *Bju*bZIP proteins and *Arabidopsis thaliana* bZIP proteins for phylogenetic analysis. Align the domain sequences using MAFFT’s linsi function, then trim them with TrimAl at 30% amino acid conservation. Construct phylogenetic trees using IQ-TREE3 based on the maximum likelihood (ML) method, selecting the optimal Le and Gascuel (LG) model for tree construction (settings: iqtree3 -s bzip_trimal.mafft -bb 1000 -redo -alrt 1000 -m LG). Finally, the phylogenetic tree of the *bZIP* gene family was refined and visualized using the EvolView web server (https://www.evolgenius.info/evolview/) (Subramanian et al., 2019).

### Sequence analysis and structural characterization

The identified bZIP protein sequences of tumorous stem mustard were submitted to ExPASy-ProtParam (Wilkins et al., 1999) web server (http://web.expasy.org/protparam/) to predict and analyze the physical and chemical properties of the amino acids, relative molecular mass, and isoelectric point of each protein. The conserved motif sequences in tumorous stem mustard bZIP protein sequences were searched using the MEME SUITE (http://meme-suite.org/tools/meme) (Bailey et al., 2015), which identified 10 motifs. The predicted motif width range was 6–50 amino acid residues, with the rest of the parameters set to default. The gene structure of the bZIP proteins in tumorous stem mustard was visualized using the Gene Structure Display Server 2.0 (http://gsds.cbi.pku.edu.cn/, GSDS) (Hu et al., 2015), which facilitated the display of exons, coding sequences (CDS), introns, and other structural elements. The integration of the phylogenetic tree of the BjubZIP transcription factor proteins, motif sequence prediction, and gene structure was facilitated by the TBtools software (Chen et al., 2020).

### Secondary structure analysis of bZIP proteins and *Bju*bZIP protein–protein interaction network prediction

The secondary structure of the bZIP protein sequences of all identified tumorous stem mustard samples was predicted and analyzed using SOPMA (Geourjon & Deleage, 1995) (https://npsa.lyon.inserm.fr/cgi-bin/npsa_automat.pl?page=/NPSA/npsa_sopma.html). The predicted structural components included α-helices, extended strands, β-turns, and random coils. A protein-protein interaction (PPI) network was constructed using the STRING database (https://cn.string-db.org/) (Szklarczyk et al., 2023) with *Brassica rapa* as the reference organism. A medium confidence score (0.400) was applied as the minimum interaction threshold to ensure statistical reliability. The network was further optimized and visualized using Cytoscape v3.10.1.

### Gene localization and cis-acting element analysis

The chromosome length information of tumorous stem mustard was extracted using SAMtools (Li et al., 2009). A Perl script was compiled to obtain the location information of each *BjubZIP* gene. After organizing the acquired gene position data, the physical map of the *BjubZIP* genes on each chromosome of tumorous stem mustard was constructed using the MapGene2Chromosome website (Chao et al., 2021) (http://mg2c.iask.in/mg2c_v2.1/). The promoter DNA sequences, 1500 bp upstream of the *bZIP* genes in the tumorous stem mustard genome, were also extracted using a Perl script and submitted to the PlantCARE database (Lescot et al., 2002) (http://bioinformatics.psb.ugent.be/webtools/plantcare/html/) for cis-acting element prediction. After manual curation, the prediction results were visualized using the GSDS 2.0 website.

### Gene tandem duplication analysis

Tandem duplications of *BjubZIP* genes were identified based on Basic Local Alignment Search Tool (BLAST) homology comparison combined with chromosomal location information. The identification of tandemly duplicated genes was based on the following two criteria: (1) in the case of relatively long genes, the alignment coverage of the two genes is more than 70% and the sequence similarity is more than 70% (Vatansever et al., 2016); (2) the physical distance between the two genes on the chromosome are less than 100kb. Genes that met these criteria were manually screened and marked in red on the chromosome physical map. The collinearity analysis of gene duplications was performed using MCScanX (Wang et al., 2012). The main configuration file, chromosome information file, collinear gene and block information file, and annotation file were prepared to illustrate collinearity relationships among genes.The collinearity analysis results were visually presented using Circos (http://circos.ca/) (Krzywinski et al., 2009) drawing software, executed *via* the Bio-Linux system command line.

### Gene ontology (GO) annotation

The functional annotation of *Bju*bZIP protein sequences and the analysis of annotation data were performed on Blast2GO (http://www.blast2go.com) (Gotz et al., 2008) software. Referring to the methods from the treatise, the amino acid sequences of *BjubZIP* s were imported into Blast2GO software to execute the following four steps: (i) running BLASTp program against the non-redundant protein sequence database (Nr) of NCBI, (ii) mapping and retrieving of the Gene Ontology (GO) terms associated with the BLASTp results, (iii) annotating the GO terms associated with each query to determine the relationship between the sequences and known protein function, and (iv) performing GO slim analysis to simplify the GO annotation results and classify all *Bju*bZIP protein annotations into designated GO functional categories. The outputs of Blast2GO software define three GO classification categories: cellular components, biological processes, and molecular functions.

### Prediction of miRNAs targeting the *BjubZIP* genes

Predicting miRNA-regulated gene targets is essential for understanding the functions of miRNAs. Pre-miRNA sequences from previously reported studies were downloaded from the miRBase website (http://www.mirbase.org) (Griffiths-Jones et al., 2008) and the Plant MicroRNA Database (https://bioinformatics.cau.edu.cn/PMRD/) (Zhang et al., 2010) were used for prediction of miRNAs targeting the *Bju*bZIP genes. In this study, the web-based psRNATarget Server (https://www.zhaolab.org/psRNATarget/analysis) (Dai, Zhuang & Zhao, 2018) was used with default parameters to identify the putative target sites of *Brassica juncea* miRNAs by aligning all known plant miRNAs with the assembled transcripts of *Bju*bZIP genes.

### Gene expression differential display

RNA-sequencing (RNA-seq) data from our previous research were downloaded from the National Center for Biotechnology Information Sequence Read Archive database (http://www.ncbi.nlm.nih.gov/sra/). The large-leaf mustard mutant stems at 22 weeks (without puffed stems, SRX108496) were used as the control group. Tumorous stem mustard was sown, and samples were collected at different time points for the experimental group: 18 weeks (before stem expansion, YA1_0, SRX108498), 20 weeks (one week before stem expansion, YA2_0, SRX108499), 22 weeks (one week after stem expansion, YA3_0, SRX108500), and 25 weeks (one month after stem expansion, YA4_0, SRX108501). Sequencing was performed using the Illumina HiSeq^TM^ 2000 platform. Each sample had three biological replicates, providing robust data for statistical analysis. Gene expression levels of individual genes were quantified using reads per kilobase of transcript per million (RPKM) values. We used a false discovery rate of < 0.001 and an absolute value of the log 2 ratio of > 1 as the threshold for judging the significance of the gene expression differences. The transcriptome sequencing results of the experimental and control groups were compared to identify differentially expressed *BjubZIP* genes during the stem expansion of tumorous stem mustard.

### Real-time fluorescent quantitative PCR of the *bZIP* gene during stem development

Gene expression profiles of BjubZIP in young leaves under high- and low-temperature treatments were analyzed as previously described (Jiang et al., 2021). Briefly, seeds were sown in a meteorite-to-soil mixture (2:1) and grown at 25 ^∘^C under a 12-h light/dark cycle. After five weeks, uniformly developed seedlings were exposed to high-temperature (12 h at 39 ^∘^C/12 h at 25 ^∘^C) or low-temperature (12 h at 5 ^∘^C/12 h at 2 ^∘^C) conditions for 0, 6, 12, 24, and 48 hours. Samples were collected at each time point, immediately frozen in liquid nitrogen, and stored at −70 ^∘^C for subsequent RNA extraction. Total RNA was isolated from whole seedlings of *Brassica juncea var. tumida* using the Plant MiniBEST RNA Extraction Kit (TaKaRa, Dalian, China). cDNA was synthesized with the PrimeScript^TM^ RT Kit and gDNA Eraser (TaKaRa). Quantitative real-time polymerase chain reaction (qRT-PCR) was conducted on a Bio-Rad iQ5 system using SYBR®Prime qPCR Set (Fast HS) (BIOGROUND, Chongqing, China) with 14 gene-specific primer pairs (Table 1).The thermocycling conditions were as follows: 94 ^∘^C for 20 s; 40 cycles at 94 ^∘^C for 10 s, 60 ^∘^C for 20 s, and 72 ^∘^C for 30 s. The *Bju*Actin gene (*BjuB012485*) was used as an internal reference (He et al., 2020). Three biological replicates were conducted for each gene, with each replicate containing three technical repeats.

**Table 1:** Primers for *bZIP* members selected for qRT-PCR.

Gene name	Forward primer sequence (5′-3′)	Reverse primer sequence (5′-3′)
*BjuB012485* (Actin)	GAGCTGAAAGATTCCGTTGC	AGTGCGGTGATCTCTTTGCT
*BjuA005478* (bZIP)	GAGAGCGAGGAGGAGGAGTT	CCTCGCTTGTTGTGCAGATA
*BjuB009536* (bZIP)	CAGCACAGCAAGCAAGAGAG	TGCATCAGCGTTAGAACCAC
*BjuA044741* (bZIP)	GGAAATCTCTGGATCGGTGA	CTCTCTTGCTTGCTGTGCTG
*BjuA041107* (bZIP)	ACCATCAAGCAGCGAGAGAT	CCTCTGACCTTCTCCGACAG
*BjuB009816* (bZIP)	ACCATCAAGCAGCGAGAGAT	CCTCCGACCGTTATCTGTGT
*BjuA018972* (bZIP)	GCGTCTTTAGTGGTGGTGGT	CCGCCGTTATTAGCATTGTT
*BjuB041699* (bZIP)	GCAGGCATATACGGTGGAGT	CTCGGGTTCCTCATCAATGT
*BjuB045319* (bZIP)	CGGCCTATGAACAACAACCT	GCGCCTGTTTTTCTTACTCG
*BjuA003230* (bZIP)	TCTTGGTGAGAGCTGGTGTG	CGGCTGAGTTTGAGTTGTCA
*BjuA040338* (bZIP)	AGGGACAAGTAGCGCTGAAA	CTCAGCTTCCAGTTCCAAGG
*BjuA025542* (bZIP)	AGATTGCTGCCAAAGATGCT	CACCTCTTATCCCAGGACCA
*BjuA025558* (bZIP)	GCGGTCACTTCGGTAATGAT	CACCTCTTATCCCAGGACCA
*BjuA012577* (bZIP)	GTTCCTCTTCTTCCCCCAAG	TTTGGGTCTGGTCAATCTCC

## Results

### Genome-wide identification of the *BjubZIP* transcription factor family

In the whole genome, 414 *bZIP* transcription factor proteins were screened by HMMER software search through 3 bZIP hidden Markov files which were obtained by the Pfam database. After removing redundant transcripts, the remaining sequences were uploaded to NCBI CDD, Pfam, and SMART databases for domain identification, and 153 protein sequences were eventually determined to be tumorous stem mustard bZIP proteins. Subcellular localization results of the BUSCA website indicated that 13 tumorous stem mustard bZIP proteins were located in the chloroplast, *Bju*A044741 was located in the chloroplast outer membrane, *Bju*A033921, *Bju*A015073, and *Bju*A017309 were located in the inner membrane system, whereas the remaining 136 were located in the nucleus.

As shown in Table S1, the average length of the amino acid sequence of *bZIP* transcription factor gene family members of tumorous stem mustard is 280 residues. Among them, the protein sequence of *Bju*A002075 was up to 680 aa; whereas the protein sequences of *Bju*A041565 and *Bju*B010770 were merely 120 aa. ExPASy-ProtParam website analysis demonstrated that the isoelectric point of bZIP proteins in tumorous stem mustard ranged from 4.94 to 10.01, and the molecular weight was between 13.91 and 74.67 kDa.

### Secondary structure of bZIP proteins and *Bju*bZIP protein–protein interaction network prediction

The physical structure of proteins plays an important role in their physiological and biochemical functions. Therefore, we predicted and analyzed the secondary structures of all stem tumour mustard bZIP proteins by SOPMA. The results showed in Table S2 that among all stem tumour mustard bZIP proteins, the largest proportion of proteins contained random coils (26.80–85.06%), followed by proteins containing α-helices (11.69–73.13%), extended strands (0.00–12.55%) and β-turns (0.00–5.65%). The Protein-Protein Interaction (PPI) network comprised 83 protein nodes and 116 edges, indicating a highly interconnected regulatory framework among *Bju*bZIP proteins (Fig. S1). Notably, *Bju*B009816 and *Bju*B009536 exhibited the highest degrees (13) and were both associated with the circadian rhythm in plants. In addition, proteins including *Bju*O004809, *Bju*O010363, *Bju*B028919, *Bju*B022206, *Bju*B016538, *Bju*B046236, *Bju*B009536, *Bju*B019686, *Bju*B009816, *Bju*B023599, *Bju*A033921, *Bju*B023986, *Bju*B008492, and *Bju*A005478 were mainly enriched in biological processes related to the positive regulation of transcription, DNA-templated.

### Classification and phylogenetic analysis of the *Bju*bZIP transcription factor family

**Figure 1:**
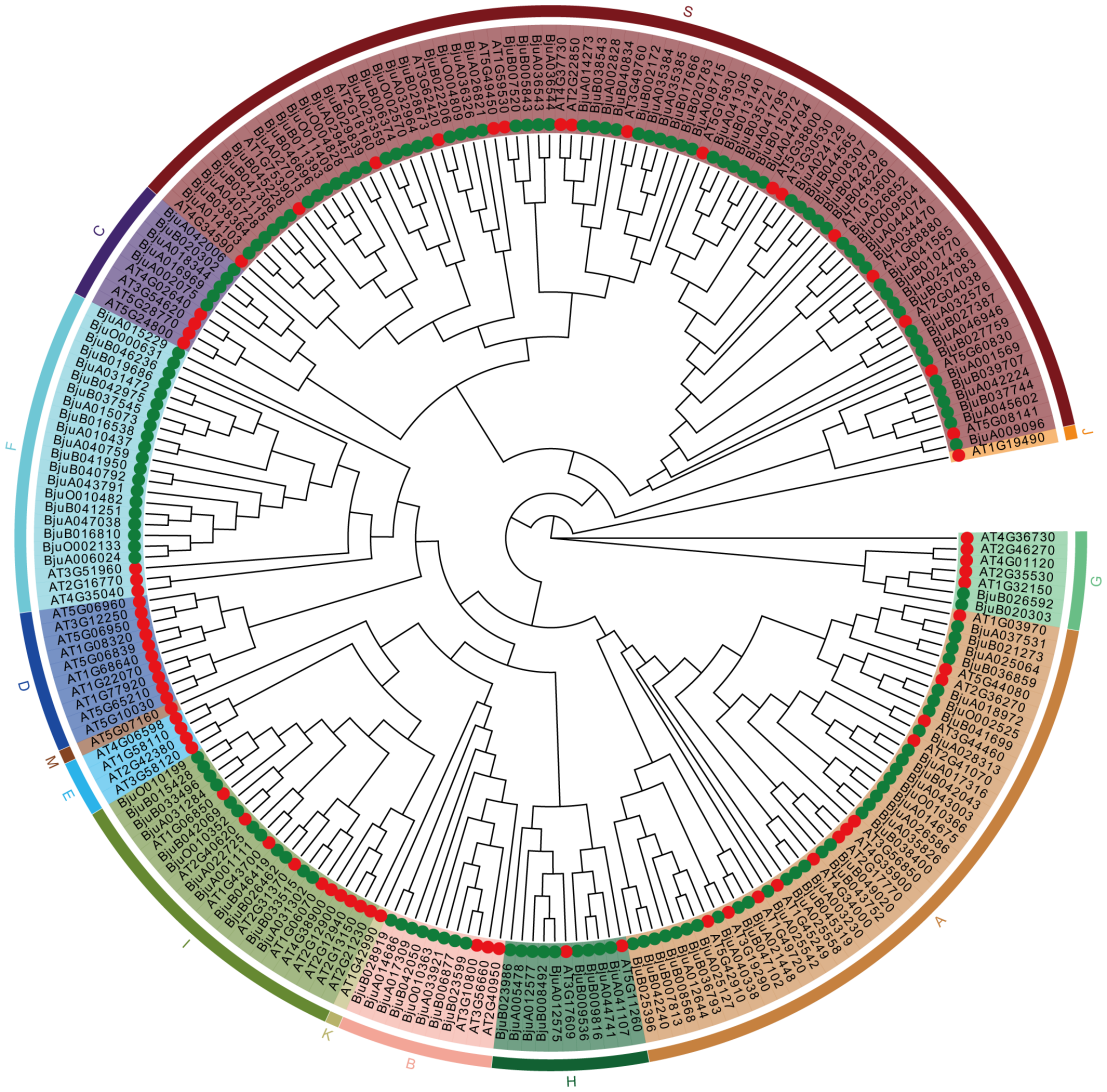
The phylogenetic tree of *bZIP* proteins between *Arabidopsis thaliana* and tumorous stem mustard. A phylogenetic tree was constructed using the conserved bZIP domain sequences of proteins from *Arabidopsis thaliana* and tumorous stem mustard. The proteins were classified into 13 subfamilies (S, A, F, I, H, B, C, G, M, K, J, D and E). Different colors represent distinct subfamilies.

A comparison was conducted between 153 confirmed BjubZIP protein domain sequences and 75 published *Arabidopsis At*bZIP protein domain sequences. Referring to the predecessor’s taxonomic criteria for *Arabidopsis At*bZIP transcription factor protein (Droge-Laser et al., 2018), this study divided the sequences involved in the construction of the tumorous stem mustard bZIP protein evolution tree into 13 categories, namely A, B, C, D, E, F, G, H, I, S, J, K, and M. As can be seen from Fig. 1, Among the 13 subfamilies, the numbers of *Bju*bZIP genes were 32, 8, 5, 0, 0, 20, 2, 9, 12, 65, 0, 0, and 0, respectively. Subfamily S contained the largest number of members (65), followed by subfamilies A (32) and F (20). In contrast, only a few proteins were assigned to subfamilies G (2) and C (5). No members were identified in subfamilies M, K, J, D, or E.

### Gene structure and amino acid sequence conservative domain analysis of *Bju*bZIP proteins

In this study, 10 amino acid motifs with different arrangements were discovered in the bZIP family members of tumorous stem mustard, suggesting that the protein structure of bZIP family members may possess extensive domain variation. In this study, the MEME website served to predict the motif composition and site distribution of tumorous stem mustard *Bju*bZIP proteins. The 10 motifs with the highest probability of occurrence of the amino acid conserved domain and the most sites were searched out, with the sequence length ranging from 21 to 41 amino acid residues, and the sequence identification map of corresponding motifs was made, which results are displayed in Table S3. The results showed that motifs 1 and 2 were the most conservative among the 10 predicted *Bju*bZIP protein motifs of tumorous stem mustard, and they were distributed in almost all *Bju*bZIP proteins. In addition, all predicted motifs are identified only once in each *Bju*bZIP protein sequence. Despite great differences among different subfamilies, at the phylogenetic tree level, closely related proteins often contain the same or similar motif structure, for instance, the protein sequences of *Bju*A017316, *Bju*B042043, *Bju*A043003, *Bju*O010366, *Bju*A014675, and *Bju*B038400 contain six identical motifs. Scanning the sequence logo corresponding to motif2, it can be found that the submitted *Bju*bZIP protein sequences conform to the characteristic that one leucine or other hydrophobic amino acid every six amino acid residues.

As shown in Fig. S2, the number of introns in the *BjubZIP* gene ranges from 0 to 12, and 68 sequences without introns, account for 44.4%. Only one sequence (accounting for 6.5%) contains a single intron, and the rest of the *BjubZIP* genes contain two or more introns. The number of introns varied greatly in different subfamilies, those without intron sequences generally belonged to the S subfamily and partly pertaining to A subfamily.

### Chromosome location and cis-acting element analysis of *BjubZIP* genes

Figure S3 manifested the distribution information of the *BjubZIP* gene on 10 chromosomes of the A sub-genome and eight chromosomes of the B sub-genome of tumorous stem mustard. The detailed position of the *BjubZIP* gene on the chromosome can be seen in Table S1, and the length of the chromosome can be estimated from Fig. S3 the scale on the left. Except that 13 identified *BjubZIP* genes were not located on specific chromosomes, the remaining 140 *BjubZIP* genes were distributed between 3 and 13 on each chromosome. Thereinto, chromosome A10 had the least number of genes containing only three. However, A09 and B02 contain 13 genes, which were the two chromosomes with the most *BjubZIP* gene distribution. The *BjubZIP* gene on the A09 chromosome was evenly distributed, on the contrary, *BjubZIP* genes on the B02 chromosome were intensively distributed at the tail end. The two homologous chromosome groups of tumorous stem mustard respectively contain 71 and 69 *BjubZIP* genes, implying that there is no obvious differentiation in the A sub-genome and B sub-genome of the content of the *bZIP* gene.

The amplification of the gene family and the evolution mechanism of the genome mainly rely on gene repeat events, in which the main duplication types are tandem duplications and fragment duplications (Cannon et al., 2004). The gene cluster is composed of two adjacent related genes repeatedly generated or a large number of identical genes arranged in series, belonging to the amplification product of a common ancestor gene, distributed in a relatively concentrated region on the chromosome. Among the 140 *Bju*bZIP genes located on each chromosome in tumorous stem mustard, 28 genes have been identified as tandem repeat genes accounting for 20%, highlighted in red in Fig. S3, which form 13 gene clusters on chromosomes and play a crucial role in the process of gene family amplification.

The cis-acting elements refer to a specific DNA sequence connected in series with a structural gene and are the binding site of a transcription factor, which regulates the precise initiation and transcription efficiency of gene transcription by binding with transcription factors (trans-acting factors). Employing the promoter prediction database Plant CARE, 107 cis-acting elements were forecasted by the promoter DNA sequence of 1500bp upstream of tumorous stem mustard *BjubZIP* genome. Except for TATA-box, CAAT-box, GC-motif, and other basic acting elements, it also included a variety of cis-acting elements related to growth and development regulation, stress induction, photoresponse regulation, hormone response, *etc*. As shown in Fig. S4, this study selected a total of 11 cis-acting elements such as circadian rhythm control elements (circadian), meristem expression elements (CAT-box), auxin response (TGA-element, AuxRR-core), gibberellin response elements (GARE-motif, P-box), abscisic acid response (ABRE), anaerobic induction (ARE), defense and stress response (TC-rich repeats), low-temperature response (LTR), drought induction(MBS), salicylic acid response (TCA-element, SARE), Methyl Jasmonate response (TGACG-motif), hypoxia-specific induction element (GC-motif), and light response regulation elements (3- AF1 binding site, AAAC-motif, ACE, G-box, GT1-motif, Sp1, MRE, *etc*.), displaying on the GSDS 2.0 website. From the above results, the cis-acting elements related to light response were present in almost all promoter regions of the *bZIP* genes of tumorous stem mustard, with an average of 4.15 per *bZIP* gene, hinting that *bZIP* gene expression of tumorous stem mustard may be under the control of the regulation of light response.

### Tandem duplication and fragment duplication of tumorous stem mustard of *BjubZIP* genes

Analyzing the collinear relationship between the genomes of species is of great theoretical and practical significance for understanding the origin, evolution, differentiation, and classification of crops and fostering excellent strains. Fig. S5 investigated the collinear relationship between genes in the tumorous stem mustard genome, reflecting the doubling information of each chromosome as well, which exhibited a total of 155 pairs of *BjubZIP* genes that have a collinear relationship. Thereinto, the gray region represents the collinear relationship among all genes in the whole genome of the tumorous stem mustard, meanwhile, the green line represents the doubling of chromosomes and gene duplication that occurs between the *BjubZIP* genes of the tumorous stem mustard. *BjuA029457* & *BjuA027015*, *BjuB047306* & *BjuB032115*, *BjuB027387* & *BjuB027387*, *BjuB008568* & *BjuB007813*, these four pairs of genes with the collinear relationship were distributed on the same chromosome, while *BjuA001569* & *BjuB037744*, *BjuA026586* & *BjuB038400 etc* genes with collinear relationship were distributed on different chromosomes.

### Gene Ontology annotation

**Figure 2:**
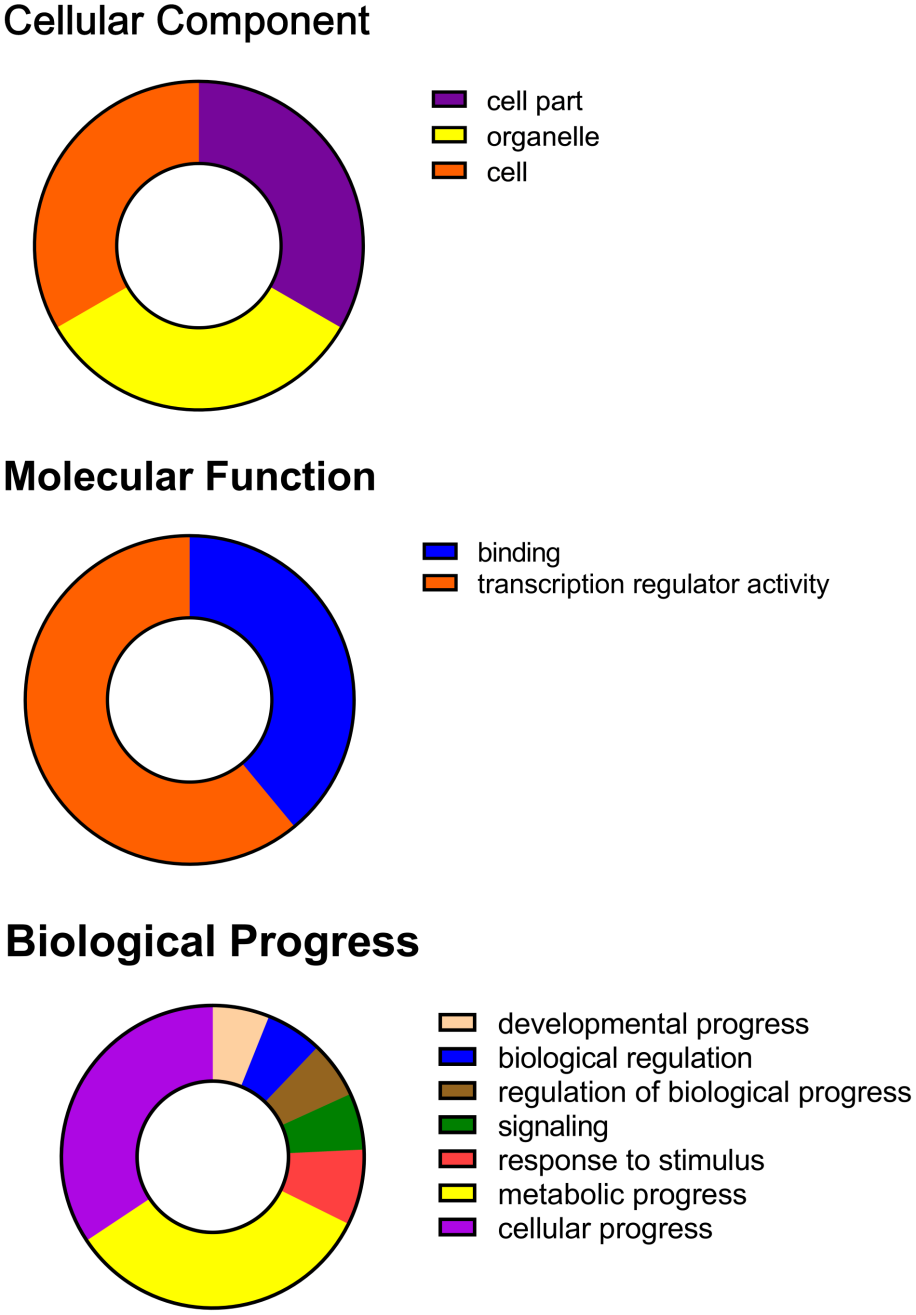
Gene Ontology (GO) distributions for the bZIP proteins. GO annotation of bZIP proteins based on three main categories: cellular component, molecular function, and biological process. The donut charts show the distribution of bZIP proteins in each GO category.

The GO slim analysis conducted through Blast2Go software has shown the putative participation of 153 *Bju*bZIP proteins in diverse biological processes in Table S4. Of the seven categories of biological processes predicted by Blast2Go software, predominant *Bju*bZIPs acted on the cellular process (∼34%), followed by the metabolic process (∼33%). Regarding molecular function, about 93 (∼39%) *Bju*bZIP proteins showed transcription factor activity, which may correlate with the abiotic stress tolerance behavior of tumorous stem mustard. In addition, molecular function prediction suggested that about 148 (∼61%) *Bju*bZIP proteins were evidenced to participate in transcription regulator activity, which concords with the molecular role of bZIP proteins as transcription factors in regulating the transcription process through auxiliary protein-protein interaction. Cellular component prediction discovered that *Bju*bZIP proteins are localized in the cell, cell part, and organelle, each accounting for one-third (Fig. 2; Table S3). The Gene Ontology was defined by the Blast2Go software by the following three categories: (i) biological processes, (ii) molecular functions, and (iii) cellular components.

### Identification of miRNAs targeting *Bju*bZIP transcripts

Referring to the scoring schema reported in miRU (Zhang, 2005), we evaluated the complementarity between miRNA and their target transcript. The most important parameters were taken into consideration for the identification of targets, namely the maximum expectation, which is the threshold of the score. If scores were greater than the threshold, the miRNA/target site pair ought to be discarded. Additionally, the default cut-off threshold was adjusted to 3.0.

A total of 85 *BjubZIP* genes targeted by 169 plant miRNAs were identified in tumorous stem mustard by psRNATarget Server. Table S5 shows the target miRNA corresponding to the *BjubZIP* gene and the maximum expectation values. The green square represents the Latin name of the species. Nevertheless, there was still a large number of plant miRNAs that could not indicate any gene targets. Among the target genes, *BjubZIP101* and *BjubZIP094* were the most abundant transcripts, which were targeted by almost 40 plant miRNAs. What’s more, the transcripts of 47 *BjubZIP* genes could indicate the corresponding targeted miRNA sequences.

### Differential expression of *BjubZIP* genes during stem expansion

Combined with the results of the sequencing of the tumor stem mustard transcriptome (Sun et al., 2012) in this laboratory, this study used the BLAST program to search for 153 *BjubZIP* genes. The experiment result expressed that 130 of them were expressed in the stems of tumorous stem mustard, indicating that 130 *BjubZIP* genes were involved in the regulation of stem development. Comparing the experimental group with the control group (DY_0), the expression level of genes like *BjubZIP001*, *BjubZIP022*, *BjubZIP105*, and *BjubZIP144* gradually increased as the stems expanded. On the contrary, that *BjubZIP017*, *BjubZIP024*, *BjubZIP039*, *BjubZIP045*, *BjubZIP047*, *BjubZIP054*, *BjubZIP069*, *BjubZIP074*, *BjubZIP075*, *BjubZIP081*, *BjubZIP085*, *BjubZIP101*, and *BjubZIP131* etcetera genes were gradually down-regulated after the growth of the stem, suggesting that these *BjubZIP* genes played a critical role in the stem expansion of the tumorous stem mustard. The detailed expression profile of *BjubZIP* genes of different development of the stem in tumorous stem mustard was displayed in Fig. S6.

### Gene expression analysis under temperature stress

**Figure 3:**
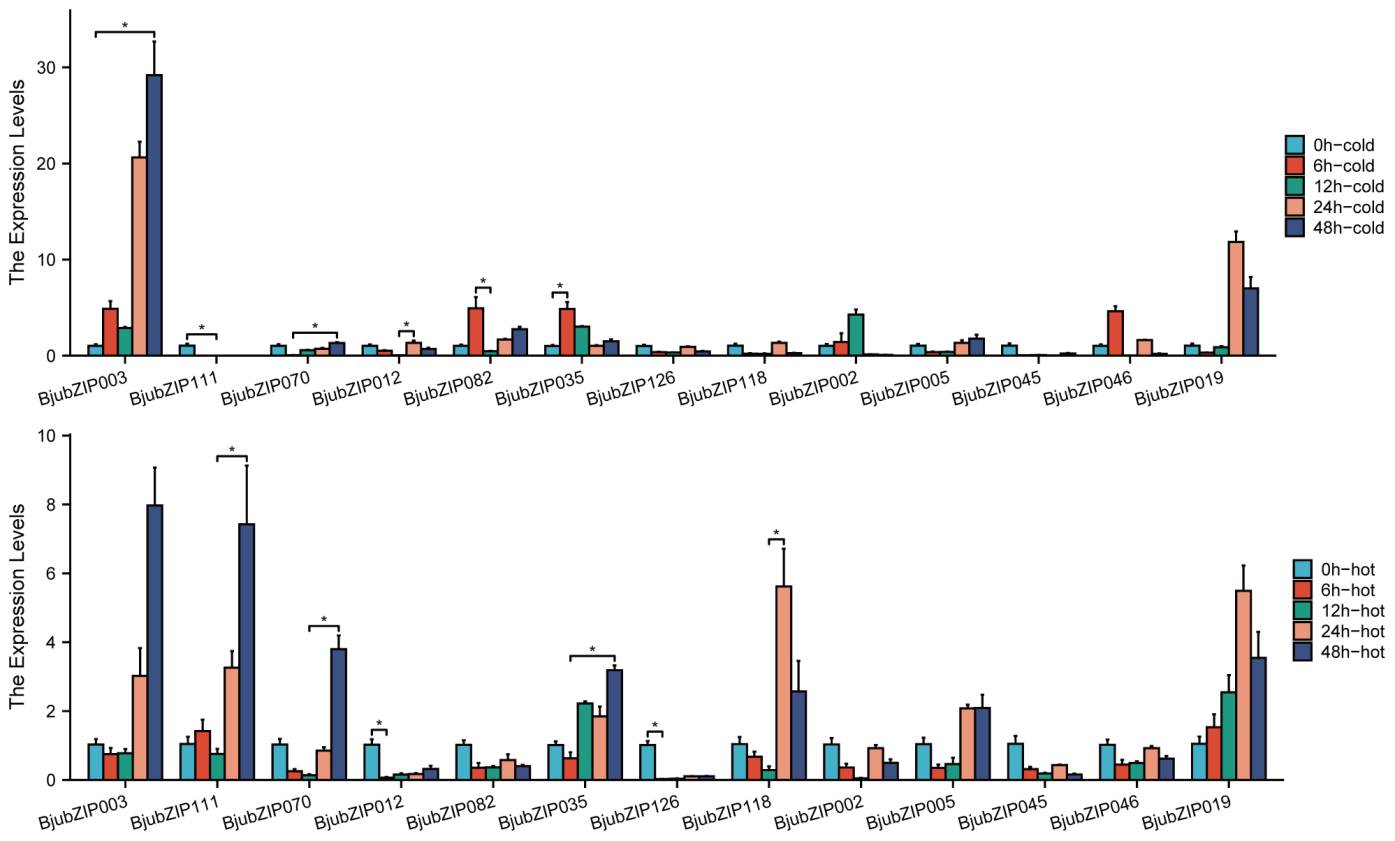
Expression levels of *BjubZIP*s under cold and hot treatment by qRT-PCR. qRT-PCR analysis of selected *BjubZIP* genes exposed to cold (top) and heat (bottom) stress at 0 h, 6 h, 12 h, 24 h, and 48 h. Statistical significance was determined using the Kruskal-Wallis test followed by Dunn’s post hoc test. Asterisks indicate significant differences (^∗^*P* < 0.05).

To further investigate the responses of *BjubZIP* genes to abiotic stress, 13 candidates enriched in GO terms such as “response to temperature stimulus”, “response to UV-B”, “response to water deprivation”, and “response to heat” were selected for qRT-PCR analysis under cold and heat treatments (Fig. 3). The results revealed diverse, time-dependent, and stress-specific expression patterns. Under heat stress, most genes were significantly up-regulated. In particular, the transcript levels of *BjubZIP111* and *BjubZIP070* increased progressively with time, reaching significantly higher levels at 48 h compared with 12 h. *BjubZIP118* was markedly induced at 24 h, whereas *BjubZIP012* and *BjubZIP126* were significantly down-regulated as early as 6 h, suggesting a role in early inhibitory responses. Under cold stress, *BjubZIP003* and *BjubZIP070* were strongly induced at 48 h, while *BjubZIP035* showed a rapid up-regulation at 6 h. In contrast, *BjubZIP082* was significantly down-regulated at 12 h. Notably, *BjubZIP111* was consistently down-regulated during cold treatment, in sharp contrast to its induction under heat stress.

## Discussion

After genome-wide analysis, 153 *Bju*bZIP proteins were identified, which were fewer than those of *Brassica napus* (247 after removal of redundancy)(Zhou et al., 2017), and slightly more than those of the diploid cabbage *Brassica oleracea (2n* = *18, CC)* in cruciferous crops (119 after redundancy removal) (Hwang et al., 2016). However, the number of proteins identified in cruciferous crops exceeds that in the model organism *Arabidopsis thaliana* (75 after redundancy removal). Subcellular localization predictions indicate that nearly 90% of bZIP proteins were localized in the nucleus, aligning with previous findings across plant species. For instance, in *Aquilaria sinensis*, Green Fluorescent Protein (GFP) fluorescence signals from *pAsbZIP14*-GFP and *pAsbZIP41*-GFP were predominantly observed in the nucleus (Zhang et al., 2023). Similarly, *BsbZIP13* in *Bletilla striata* (Zhou et al., 2023), *PhebZIP47* and *PhebZIP126* in *Phyllostachys edulis* (Pan et al., 2019), *BrABI5a* and *BrABI5b* in Chinese cabbage (*Brassica rapa*) (Bai et al., 2016) were confirmed to be nuclear localized by subcellular localization experiments. In addition, *CibZIP38*, *CibZIP55*, and *CibZIP43* in *Carya illinoinensis* (Jiang et al., 2023) and *CsbZIP55* and *CsbZIP65* in cucumber *(Cucumis sativus* L.) were also nuclear localized (Hua et al., 2023). As expected, the nucleus serves as the control center for cellular heredity and metabolism. Unsurprisingly, this further supports the role of bZIP proteins as transcription factors regulating the expression of key genes in plants. Furthermore, the physicochemical properties of bZIP transcription factor family members in tumorous stem mustard appear to be highly diverse, suggesting their potential functional versatility.

By comparing *BjubZIP* genes with published *bZIP* gene family members from different species, the evolutionary history, origin, and function of those with unclear characteristics can be effectively inferred. In tumorous stem mustard, bZIP proteins are mainly distributed in several subfamilies such as S and A, but are absent in five subfamilies including D and E. We speculate that the *BjubZIP* subfamily genes not found above may have been lost during the evolution of the tumorous stem mustard genome.

The number of introns varies widely among subfamilies. Our study showed that 33.7% of the *BjubZIP* genes were intronless, which contrasts with previously reported studies in *Stevia rebaudiana* (19%) (Wu et al., 2023), rice (15.3%) (Nijhawan et al., 2008), tomato (17.4%) (Li et al., 2015) and poplar (22%) (Zhao et al., 2021). It has been shown that the S group consists of the largest 17 bZIP clusters, which are typically intronless and encode small TF proteins of approximately 20 kDa in size (Droge-Laser et al., 2018). This aligns with our fingding in tumorous stem mustard, where most of the bZIP proteins distributed in the subfamily S are intronless. Therefore, we speculate that the loss and acquisition of exons occurred during the evolution of the *BjubZIP* subfamily. The loss and acquisition of exons and introns serve as a mechanism for the diversification of polygenic families, which may result from chromosome rearrangement and potentially lead to the generation of functionally distinct paralogous genes. Under normal circumstances, genes with no introns or only a few introns tend to have low expression levels in plants, which differs significantly from the pattern observed in animals (Shabalina et al., 2010). However, when dealing with some internal or external stimuli, these genes can perform a rapid increase in expression. For instance, the gene with only two introns (*BjuA012577*) exhibits low expression levels in many tissues but is significantly upregulated under heat shock conditions.

The cis-acting elements refer to a specific DNA sequence connected in series with a structural gene and are the binding site of a transcription factor, which regulates the precise initiation and transcription efficiency of gene transcription by binding with transcription factors (trans-acting factors). After the prediction analysis, we found the cis-acting elements related to light response were present in almost all promoter regions of the *bZIP* genes of tumorous stem mustard, with an average of 4.15 per *bZIP* gene, hinting that *bZIP* gene expression of tumorous stem mustard may be under the control of the regulation of light response. This is consistent with previous reports on the regulation of photosynthesis by *bZIP* (Droge-Laser et al., 2018). The low-temperature response elements were absent in some *bZIP* genes, for instance, *BjubZIP126* and *BjubZIP045*, implying that certain *bZIP* genes in tumorous stem mustard may regulate cold stress responses through alternative pathways.

Analyzing the collinear relationship between the genomes of species is of great theoretical and practical significance for understanding the origin, evolution, differentiation, and classification of crops and fostering excellent strains. Through the Circos diagram, we found that the *bZIP* gene family in tumorous stem mustard had undergone amplification and genome evolution due to the tandem duplications and fragment duplications. Chromosome doubling and gene duplication existed in a large number of genes between A and B subgenomes, resulting in the number of the *bZIP* gene family in tumorous stem mustard much larger than that of model organisms such as *Arabidopsis thaliana* and rice.

The *bZIP* genes are involved in various abiotic stresses in plants (Yang et al., 2025), including low, drought, and high-temperature stresses, as well as biotic stresses such as diseases and pathogens (Gai et al., 2022), and various hormone-induced processes. *LchibZIP* genes were found to regulate cold stress, especially *LchibZIP4* and *LchibZIP7*, in *Liriodendron chinense* (Li et al., 2022). A basic leucine zipper domain (bZIP) transcription factor (TF) of kiwifruit (*Actinidia eriantha Benth*.), *AcePosF21*, was triggered by cold and has been shown to be involved in the regulation of kiwifruit AsA biosynthesis and defense responses against cold stress (Liu et al., 2023). It has been shown that heat stress in *Arabidopsis* and maize seedlings activates the splicing of mRNA for the transcription factor *bZIP60* (Deng et al., 2011; Li, Humbert & Howell, 2012). *Po*bZIPs have been identified as key players in the heat stress tolerance pathway of the flat mushroom (*Pleurotus ostreatus*). Specifically, *PobZIP3* has been shown to mitigate heat stress-induced mycelial damage, enhance the fungus’s capacity for growth recovery, and expedite protoplast emergence and fruit production (Zhou et al., 2024).

The results of quantitative real-time PCR (qRT-PCR) analysis of selected *bZIP* genes in stem mustard revealed alterations in the expression levels of 13 *bZIP* genes in response to cold and heat stress treatments. *BjubZIP126* exhibited no cold response elements and showed a significant decrease in gene expression following 6 hours of heat treatment. Genes containing cold response elements (LTR) within their promoters, such as *BjubZIP003*, *BjubZIP070*, and *BjubZIP035*, were significantly upregulated after 24–48 hours of cold treatment, consistent with the cold response function mediated by LTR elements. Conversely, *BjubZIP082* exhibited significant downregulation after 12 hours of low-temperature exposure despite possessing an LTR element in its promoter. This suggests that cis-element presence does not necessarily induce expression, with its function potentially regulated by other transcription factors. Furthermore, ABA response elements (ABREs) are widely present in the promoters of multiple genes (*e.g. BjubZIP111*, *BjubZIP118*, and *BjubZIP005*). These genes were significantly upregulated under high-temperature treatment, indicating that ABA signalling may participate in their activation under heat stress conditions. Among the altered genes, *BjubZIP111* was down-regulated by cold but induced by heat, implying a condition-dependent regulation mediated by ABRE-related pathways.

## Conclusions

In summary, this study conducted a genome-wide analysis of the *bZIP* transcription factor family in tumorous stem mustard, identified 153 tumorous stem mustard *bZIP* genes, and predicted the physical and chemical properties, as well as the secondary structures, of different *Bju*bZIP proteins. The results of subcellular localization showed that 13 tumorous stem mustard bZIP proteins were located in the chloroplast, *Bju*A044741 was located in the outer chloroplast membrane, *Bju*A033921, *Bju*A015073 and *Bju*A017309 were located in the inner membrane system, and the remaining 136 were located in the nucleus. Approximately 90% of bZIP protein is located in the nucleus, further verifying that bZIP protein as a transcription factor regulates the expression of tissue-specific genes in individuals. The *BjubZIP* gene is distributed on the chromosomes of 18 stem tumors. We divided the bZIP proteins in Arabidopsis and stem tumors mustard on phylogenetic trees into 13 subfamilies, respectively, A, B, C, D, E, F, G, H, I, S, J, K, and M, mainly distributed in the S and A subfamilies. However, the *BjubZIP* genes in tumorous stem mustard were only distributed in A, B, C, F, G, H, I, and S subfamilies. We speculate that the subfamily genes without *Bju*bZIP protein have been lost during the evolution of the tumorous stem mustard genome. There are 28 genes identified as tandem duplications, which all play a critical role in the amplification process of gene families. The exon-intron structure of the gene and the motif arrangement of *Bju*bZIP proteins in the subfamily further support the classification of phylogenetic tree prediction.

The cis-acting element in the promoter region of about 1.5 kb upstream of the gene regulates the transcription process of the corresponding gene in response to various environmental signals, which has an important influence on the growth and development of the plant. In this study, the Plant CARE database predicted that there were 107 species of 16,653 cis-acting elements in the promoter region of tumorous stem mustard *bZIP* gene, primarily including cis-acting elements related to growth and development regulation, hormone response, stress induction, photoresponse regulation, tissue-specific expression, and other processes, which further confirmed the function of tumorous stem mustard *bZIP* gene expression products as transcription factors. In this study, RNA-seq data were utilized to analyze the expression of *BjubZIP* genes at varying developmental stages in tumour stem mustard. A total of 130 *BjubZIP* genes were implicated in the regulation of stem development. The effects of temperature stress on 13 *BjubZIP* genes were elucidated by qRT-PCR, and the expression of the majority of the genes was found to be up-regulated. The expression of *BjubZIP111* was found to be downregulated under cold treatment and significantly up-regulated under heat treatment conditions after 48 hours of cold and heat stress treatment. In contrast, the expression of *BjubZIP082* was significantly up-regulated under cold treatment and down-regulated under heat treatment. These findings not only provide a comprehensive understanding of the *bZIP* gene family in tumorous stem mustard but also lay a valuable foundation for future studies on the molecular mechanisms of stress responses and developmental regulation in polyploid *Brassica crops*.

## Supplemental Information

10.7717/peerj.20518/supp-1 Supplemental Information 1 Protein Information of *bZIP* Gene Family in Stem Mustard

10.7717/peerj.20518/supp-2 Supplemental Information 2 The secondary structure of bZIP proteins

10.7717/peerj.20518/supp-3 Supplemental Information 3 Prediction Results of Motif in Amino Acid Conservative Domain

10.7717/peerj.20518/supp-4 Supplemental Information 4 Blast2Go annotation details of * BjubZIP* protein sequences

10.7717/peerj.20518/supp-5 Supplemental Information 5 miRNA targets predicted by psRNATarget Server

10.7717/peerj.20518/supp-6 Supplemental Information 6 Protein–protein interaction (PPI) network of *BjubZIP* proteins The PPI network of BjubZIP proteins was constructed using STRING (organism: *Brassica rapa* , interaction score ≥ 0.400) and visualized in Cytoscape v3.10.1. Nodes represent BjubZIP proteins, and edges indicate predicted functional associations.

10.7717/peerj.20518/supp-7 Supplemental Information 7 Phylogenetic tree, protein domains, conserved motifs, and gene structures of the *bZIP* gene family in tumorous stem mustard The phylogenetic tree shows the classification of BjubZIP proteins into different subfamilies. Conserved motifs (colored boxes) were identified using MEME, and protein domain annotation highlights the conserved bZIP domains. Gene structures were analyzed with GSDS, where green boxes represent exons and black lines represent introns.

10.7717/peerj.20518/supp-8 Supplemental Information 8 Physical Map of Chromosome Location of *BjubZIP* Genes The physical locations of *BjubZIP* genes are mapped onto 18 chromosomes (A01–A10, B01–B 08 ). Gene IDs are shown on the side of each chromosome, with positions (Mb) indicated on the left. Genes highlighted in red represent duplicated or tandemly clustered members.

10.7717/peerj.20518/supp-9 Supplemental Information 9 Distribution maps of cis-acting elements in the stem tumor mustard of *BjubZIP* genes Cis-acting regulatory elements identified within 1500 bp upstream promoter regions of *BjubZIP* genes in tumorous stem mustard. Different colors represent distinct types of cis-elements.

10.7717/peerj.20518/supp-10 Supplemental Information 10 Results of MCScanX analysis of stem tumor mustard *BjubZIP* genes The gray lines indicate all synteny blocks in the tumorous stem mustard genome, and the green lines indicate duplicated *bZIP* gene pairs. The chromosome number is indicated at the inside of each chromosome.

10.7717/peerj.20518/supp-11 Supplemental Information 11 Expression profile of *BjubZIP* of development of the stem in tumorous stem mustard Heatmap of standardized expression levels (Z-scores) of *BjubZIP* genes across five developmental stages (DY_0, YA1_0, YA2_0, YA3_0, YA4_0). Red indicates high expression, green indicates low expression, and yellow indicates intermediate expression.

10.7717/peerj.20518/supp-12 Supplemental Information 12 MIQE checklist

10.7717/peerj.20518/supp-13 Supplemental Information 13 Information on the bzip gene family

10.7717/peerj.20518/supp-14 Supplemental Information 14 Differentially expressed fpkm values for the bzip gene

10.7717/peerj.20518/supp-15 Supplemental Information 15 Expression analysis of bjubzip under low temperature stresses

10.7717/peerj.20518/supp-16 Supplemental Information 16 Sopma predicts protein secondary structure

10.7717/peerj.20518/supp-17 Supplemental Information 17 Raw data and code about hmmsearch, miRNA prediction, motif, subcellular locaalizaation prediction, exon ,cis-acting element and other data

10.7717/peerj.20518/supp-18 Supplemental Information 18 Raw data about circos

10.7717/peerj.20518/supp-19 Supplemental Information 19 Duplication gene result

10.7717/peerj.20518/supp-20 Supplemental Information 20 Ks, Ka and Ka/Ks ratios for gene pairs

## Additional Information and Declarations

### Data Availability

The following information was supplied regarding data availability: All the raw data and the code are available in the Supplemental File.

## References

[ref-1] Abe M, Kobayashi Y, Yamamoto S, Daimon Y, Yamaguchi A, Ikeda Y, Ichinoki H, Notaguchi M, Goto K, Araki T (2005). FD, a bZIP protein mediating signals from the floral pathway integrator FT at the shoot apex. Science.

[ref-2] Bai Y, Zhu W, Hu X, Sun C, Li Y, Wang D, Wang Q, Pei G, Zhang Y, Guo A, Zhao H, Lu H, Mu X, Hu J, Zhou X, Xie CG (2016). Genome-wide analysis of the bZIP gene family identifies two ABI5-like bZIP transcription factors, BrABI5a and BrABI5b, as positive modulators of ABA signalling in Chinese cabbage. PLOS ONE.

[ref-3] Bailey TL, Johnson J, Grant CE, Noble WS (2015). The MEME suite. Nucleic Acids Research.

[ref-4] Berardini TZ, Reiser L, Li D, Mezheritsky Y, Muller R, Strait E, Huala E (2015). The Arabidopsis information resource: making and mining the gold standard annotated reference plant genome. Genesis.

[ref-5] Cannon SB, Mitra A, Baumgarten A, Young ND, May G (2004). The roles of segmental and tandem gene duplication in the evolution of large gene families in *Arabidopsis thaliana*. BMC Plant Biology.

[ref-6] Chao J, Li Z, Sun Y, Aluko OO, Wu X, Wang Q, Liu G (2021). MG2C: a user-friendly online tool for drawing genetic maps. Molecular Horticulture.

[ref-7] Chen C, Chen H, Zhang Y, Thomas HR, Frank MH, He Y, Xia R (2020). TBtools: an integrative toolkit developed for interactive analyses of big biological data. Molecular Plant.

[ref-8] Chen H, Wang T, He X, Cai X, Lin R, Liang J, Wu J, King G, Wang X (2022). BRAD V3.0: an upgraded Brassicaceae database. Nucleic Acids Research.

[ref-9] Dai X, Zhuang Z, Zhao PX (2018). psRNATarget: a plant small RNA target analysis server (2017 release). Nucleic Acids Research.

[ref-10] Deng Y, Humbert S, Liu JX, Srivastava R, Rothstein SJ, Howell SH (2011). Heat induces the splicing by IRE1 of a mRNA encoding a transcription factor involved in the unfolded protein response in Arabidopsis. Proceedings of the National Academy of Sciences of the United States of America.

[ref-11] Droge-Laser W, Snoek BL, Snel B, Weiste C (2018). The Arabidopsis bZIP transcription factor family—an update. Current Opinion in Plant Biology.

[ref-12] Ellenberger TE, Brandl CJ, Struhl K, Harrison SC (1992). The GCN4 basic region leucine zipper binds DNA as a dimer of uninterrupted alpha helices: crystal structure of the protein-DNA complex. Cell.

[ref-13] Finn RD, Clements J, Eddy SR (2011). HMMER web server: interactive sequence similarity searching. Nucleic Acids Research.

[ref-14] Fujita Y, Fujita M, Satoh R, Maruyama K, Parvez MM, Seki M, Hiratsu K, Ohme-Takagi M, Shinozaki K, Yamaguchi-Shinozaki K (2005). AREB1 is a transcription activator of novel ABRE-dependent ABA signaling that enhances drought stress tolerance in Arabidopsis. The Plant Cell.

[ref-15] Gai Y, Li L, Liu B, Ma H, Chen Y, Zheng F, Sun X, Wang M, Jiao C, Li H (2022). Distinct and essential roles of bZIP transcription factors in the stress response and pathogenesis in Alternaria alternata. Microbiology Research.

[ref-16] Geourjon C, Deleage G (1995). SOPMA: significant improvements in protein secondary structure prediction by consensus prediction from multiple alignments. Computer Applications in the Biosciences.

[ref-17] Gibalova A, Renak D, Matczuk K, Dupl’akova N, Chab D, Twell D, Honys D (2009). AtbZIP34 is required for Arabidopsis pollen wall patterning and the control of several metabolic pathways in developing pollen. Plant Molecular Biology.

[ref-18] Gotz S, Garcia-Gomez JM, Terol J, Williams TD, Nagaraj SH, Nueda MJ, Robles M, Talon M, Dopazo J, Conesa A (2008). High-throughput functional annotation and data mining with the Blast2GO suite. Nucleic Acids Research.

[ref-19] Griffiths-Jones S, Saini HK, Van Dongen S, Enright AJ (2008). miRBase: tools for microRNA genomics. Nucleic Acids Research.

[ref-20] Guiltinan MJ, Marcotte Jr WR, Quatrano RS (1990). A plant leucine zipper protein that recognizes an abscisic acid response element. Science.

[ref-21] He J, He X, Chang P, Jiang H, Gong D, Sun Q (2020). Genome-wide identification and characterization of TCP family genes in Brassica juncea var. tumida. PeerJ.

[ref-22] Hu B, Jin J, Guo AY, Zhang H, Luo J, Gao G (2015). GSDS 2.0: an upgraded gene feature visualization server. Bioinformatics.

[ref-23] Hua B, Liang F, Zhang W, Qiao D, Wang P, Teng H, Zhang Z, Liu J, Miao M (2023). The potential role of bZIP55/65 in nitrogen uptake and utilization in cucumber is revealed *via* bZIP gene family characterization. Plants.

[ref-24] Hwang I, Manoharan RK, Kang JG, Chung MY, Kim YW, Nou IS (2016). Genome-wide identification and characterization of bZIP transcription factors in Brassica oleracea under cold stress. BioMed Research International.

[ref-25] Iven T, Strathmann A, Bottner S, Zwafink T, Heinekamp T, Guivarc’h A, Roitsch T, Droge-Laser W (2010). Homo- and heterodimers of tobacco bZIP proteins counteract as positive or negative regulators of transcription during pollen development. The Plant Journal.

[ref-26] Izawa TFR, Nakajima M, Shimamoto K, Chua NH (1994). The rice bZIP transcriptional activator RITA-1 is highly expressed during seed development. The Plant Cell.

[ref-27] Jakoby M, Weisshaar B, Droge-Laser W, Vicente-Carbajosa J, Tiedemann J, Kroj T, Parcy F, Ziprg b (2002). bZIP transcription factors in Arabidopsis. Trends in Plant Science.

[ref-28] Jiang L, Sun Q, Wang Y, Chang P, Kong H, Luo C, He X (2021). Genome-wide identification and characterization of NAC genes in Brassica juncea var. tumida. PeerJ.

[ref-29] Jiang N, Wang L, Lan Y, Liu H, Zhang X, He W, Wu M, Yan H, Xiang Y (2023). Genome-wide identification of the Carya illinoinensis bZIP transcription factor and the potential function of S1-bZIPs in abiotic stresses. Tree Genetics & Genomes.

[ref-30] Karin M (1990). Too many transcription factors: positive and negative interactions. New Biologist.

[ref-31] Kang L, Qian L, Zheng M, Chen L, Chen H, Yang L, You L, Yang B, Yan M, Gu Y, Wang T, Schiessl SV, An H, Blischak P, Liu X, Lu H, Zhang D, Rao Y, Jia D, Zhou D, Xiao H, Wang Y, Xiong X, Mason AS, Chris Pires J, Snowdon RJ, Hua W, Liu Z (2021). Genomic insights into the origin, domestication and diversification of Brassica juncea. Nature Genetics.

[ref-32] Krzywinski M, Schein J, Birol I, Connors J, Gascoyne R, Horsman D, Jones SJ, Marra MA (2009). Circos: an information aesthetic for comparative genomics. Genome Research.

[ref-33] Landschulz WH, Johnson PF, McKnight SL (1988). The leucine zipper: a hypothetical structure common to a new class of DNA binding proteins. Science.

[ref-34] Latchman DS (1997). Transcription factors: an overview. International Journal of Biochemistry and Cell Biology.

[ref-35] Lescot M, Dehais P, Thijs G, Marchal K, Moreau Y, Van de Peer Y, Rouze P, Rombauts S (2002). PlantCARE, a database of plant cis-acting regulatory elements and a portal to tools for in silico analysis of promoter sequences. Nucleic Acids Research.

[ref-36] Letunic I, Khedkar S, Bork P (2021). SMART: recent updates, new developments and status in 2020. Nucleic Acids Research.

[ref-37] Li D, Fu F, Zhang H, Song F (2015). Genome-wide systematic characterization of the bZIP transcriptional factor family in tomato (Solanum lycopersicum L.). BMC Genomics.

[ref-38] Li H, Handsaker B, Wysoker A, Fennell T, Ruan J, Homer N, Marth G, Abecasis G, Durbin R, Genome Project Data Processing S (2009). The sequence alignment/map format and SAMtools. Bioinformatics.

[ref-39] Li Y, Humbert S, Howell SH (2012). ZmbZIP60 mRNA is spliced in maize in response to ER stress. BMC Research Notes.

[ref-40] Li M, Hwarari D, Li Y, Ahmad B, Min T, Zhang W, Wang J, Yang L (2022). The bZIP transcription factors in Liriodendron chinense: Genome-wide recognition, characteristics and cold stress response. Frontiers in Plant Science.

[ref-41] Liao Y, Zou HF, Wei W, Hao YJ, Tian AG, Huang J, Liu YF, Zhang JS, Chen SY (2008). Soybean GmbZIP44, GmbZIP62 and GmbZIP78 genes function as negative regulator of ABA signaling and confer salt and freezing tolerance in transgenic Arabidopsis. Planta.

[ref-42] Liu X, Bulley SM, Varkonyi-Gasic E, Zhong C, Li D (2023). Kiwifruit bZIP transcription factor AcePosF21 elicits ascorbic acid biosynthesis during cold stress. Plant Physiology.

[ref-43] Mistry J, Chuguransky S, Williams L, Qureshi M, Salazar GA, Sonnhammer ELL, Tosatto SCE, Paladin L, Raj S, Richardson LJ, Finn RD, Bateman A (2021). Pfam: the protein families database in 2021. Nucleic Acids Research.

[ref-44] Nijhawan A, Jain M, Tyagi AK, Khurana JP (2008). Genomic survey and gene expression analysis of the basic leucine zipper transcription factor family in rice. Plant Physiology.

[ref-45] Pan F, Wu M, Hu W, Liu R, Yan H, Xiang Y (2019). Genome-wide identification and expression analyses of the bZIP transcription factor genes in moso bamboo (*Phyllostachys edulis*). International Journal of Molecular Sciences.

[ref-46] Savojardo C, Martelli PL, Fariselli P, Profiti G, Casadio R (2018). BUSCA: an integrative web server to predict subcellular localization of proteins. Nucleic Acids Research.

[ref-47] Shabalina SA, Ogurtsov AY, Spiridonov AN, Novichkov PS, Spiridonov NA, Koonin EV (2010). Distinct patterns of expression and evolution of intronless and intron-containing mammalian genes. Molecular Biology and Evolution.

[ref-48] Subramanian B, Gao S, Lercher MJ, Hu S, Chen WH (2019). Evolview v3: a webserver for visualization, annotation, and management of phylogenetic trees. Nucleic Acids Research.

[ref-49] Sun Q, Zhou G, Cai Y, Fan Y, Zhu X, Liu Y, He X, Shen J, Jiang H, Hu D, Pan Z, Xiang L, He G, Dong D, Yang J (2012). Transcriptome analysis of stem development in the tumourous stem mustard *Brassica juncea var. tumida* Tsen et Lee by RNA sequencing. BMC Plant Biology.

[ref-50] Szklarczyk D, Kirsch R, Koutrouli M, Nastou K, Mehryary F, Hachilif R, Gable AL, Fang T, Doncheva NT, Pyysalo S, Bork P, Jensen LJ, Von Mering C (2023). The STRING database in 2023: protein-protein association networks and functional enrichment analyses for any sequenced genome of interest. Nucleic Acids Research.

[ref-51] Talanian RV, McKnight CJ, Kim PS (1990). Sequence-specific DNA binding by a short peptide dimer. Science.

[ref-52] Toh S, McCourt P, Tsuchiya Y (2012). HY5 is involved in strigolactone-dependent seed germination in Arabidopsis. Plant Signaling & Behavior.

[ref-53] Vatansever R, Koc I, Ozyigit II, Sen U, Uras ME, Anjum NA, Pereira E, Filiz E (2016). Genome-wide identification and expression analysis of sulfate transporter (SULTR) genes in potato (*Solanum tuberosum* L.). Planta.

[ref-54] Wang Y, Tang H, Debarry JD, Tan X, Li J, Wang X, Lee TH, Jin H, Marler B, Guo H, Kissinger JC, Paterson AH (2012). MCScanX: a toolkit for detection and evolutionary analysis of gene synteny and collinearity. Nucleic Acids Research.

[ref-55] Wang J, Zhou J, Zhang B, Vanitha J, Ramachandran S, Jiang SY (2011). Genome-wide expansion and expression divergence of the basic leucine zipper transcription factors in higher plants with an emphasis on sorghum. Journal of Integrative Plant Biology.

[ref-56] Wei K, Chen J, Wang Y, Chen Y, Chen S, Lin Y, Pan S, Zhong X, Xie D (2012). Genome-wide analysis of bZIP-encoding genes in maize. DNA Research.

[ref-57] Wilkins MR, Gasteiger E, Bairoch A, Sanchez JC, Williams KL, Appel RD, Hochstrasser DF (1999). Protein identification and analysis tools in the ExPASy server. Methods in Molecular Biology.

[ref-58] Wu M, Chen J, Tang W, Jiang Y, Hu Z, Xu D, Hou K, Chen Y, Wu W (2023). Genome-wide identification and expression analysis of bZIP family genes in Stevia rebaudiana. Gene.

[ref-59] Yang J, Liu D, Wang X, Ji C, Cheng F, Liu B, Hu Z, Chen S, Pental D, Ju Y, Yao P, Li X, Xie K, Zhang J, Wang J, Liu F, Ma W, Shopan J, Zheng H, Mackenzie SA, Zhang M (2016). The genome sequence of allopolyploid Brassica juncea and analysis of differential homoeolog gene expression influencing selection. Nature Genetics.

[ref-60] Yang Y, Xu Y, Feng B, Li P, Li C, Zhu CY, Ren SN, Wang HL (2025). Regulatory networks of bZIPs in drought, salt and cold stress response and signaling. Plant Science.

[ref-61] Yang J, Zhang C, Zhao N, Zhang L, Hu Z, Chen S, Zhang M (2018). Chinese root-type mustard provides phylogenomic insights into the evolution of the multi-use diversified Allopolyploid Brassica juncea. Molecular Plant.

[ref-62] Zhang Y (2005). miRU: an automated plant miRNA target prediction server. Nucleic Acids Research.

[ref-63] Zhang H, Ding X, Wang H, Chen H, Dong W, Zhu J, Wang J, Peng S, Dai H, Mei W (2023). Systematic evolution of bZIP transcription factors in Malvales and functional exploration of AsbZIP14 and AsbZIP41 in Aquilaria sinensis. Frontiers in Plant Science.

[ref-64] Zhang Z, Yu J, Li D, Zhang Z, Liu F, Zhou X, Wang T, Ling Y, Su Z (2010). PMRD: plant microRNA database. Nucleic Acids Research.

[ref-65] Zhao K, Chen S, Yao W, Cheng Z, Zhou B, Jiang T (2021). Genome-wide analysis and expression profile of the bZIP gene family in poplar. BMC Plant Biology.

[ref-66] Zhou Y, Li Z, Xu C, Pan J, Li H, Zhou Y, Zou Y (2024). Genome-wide analysis of bZIP gene family members in Pleurotus ostreatus, and potential roles of PobZIP3 in development and the heat stress response. Microbial Biotechnology.

[ref-67] Zhou Y, Xu D, Jia L, Huang X, Ma G, Wang S, Zhu M, Zhang A, Guan M, Lu K, Xu X, Wang R, Li J, Qu C (2017). Genome-wide identification and structural analysis of bZIP transcription factor genes in Brassica napus. Gene.

[ref-68] Zhou R, Zhao G, Zheng S, Xie S, Lu C, Liu S, Wang Z, Niu J (2023). Comprehensive functional analysis of the bZIP family in Bletilla striata reveals that BsbZIP13 could respond to multiple abiotic stresses. International Journal of Molecular Sciences.

