# Supplementary figures and images for: Genome-wide systematic characterization of bZIP transcription factors and their expression profiles during stem in tumorous stem mustard
Source: PeerJ. 2026 Jan 14;14:e20518. doi: 10.7717/peerj.20518 (PMC12811965; doi:10.7717/peerj.20518)

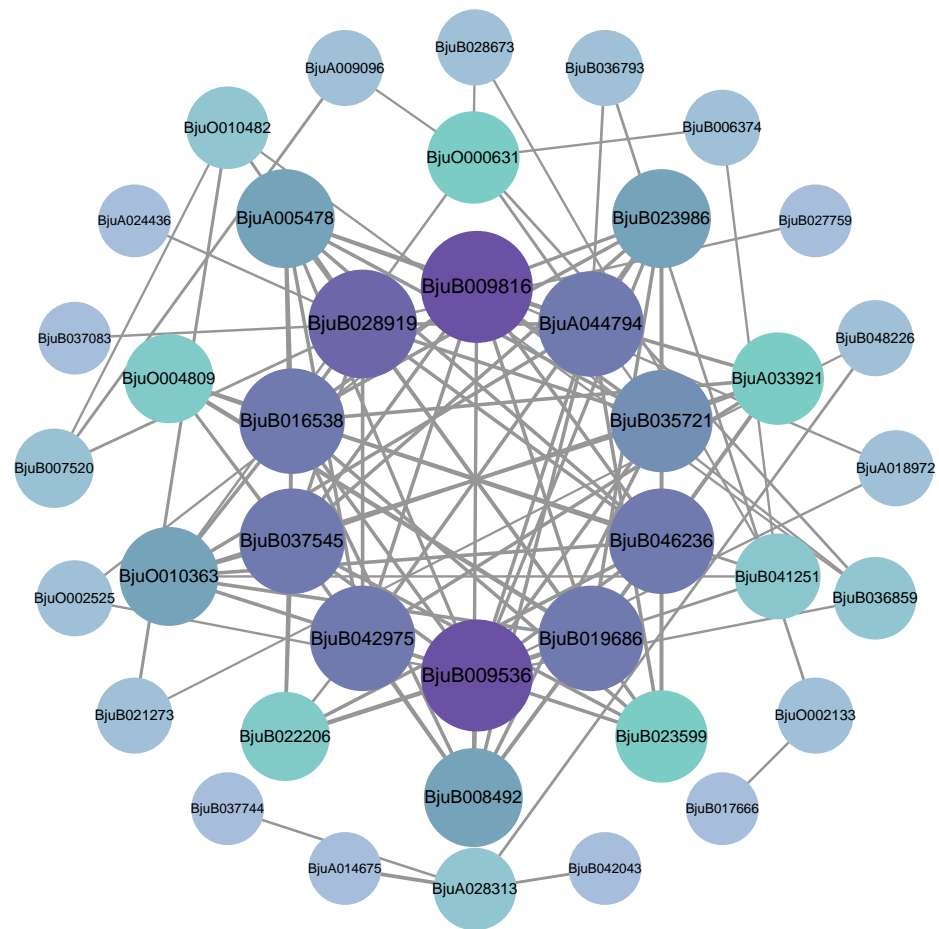

Supplement: Supplemental Information 6 — The PPI network of BjubZIP proteins was constructed using STRING (organism: Brassica rapa , interaction score ≥ 0.400) and visualized in Cytoscape v3.10.1. Nodes represent BjubZIP proteins, and edges indicate predicted functional associations. [file peerj-14-20518-s006.pdf]

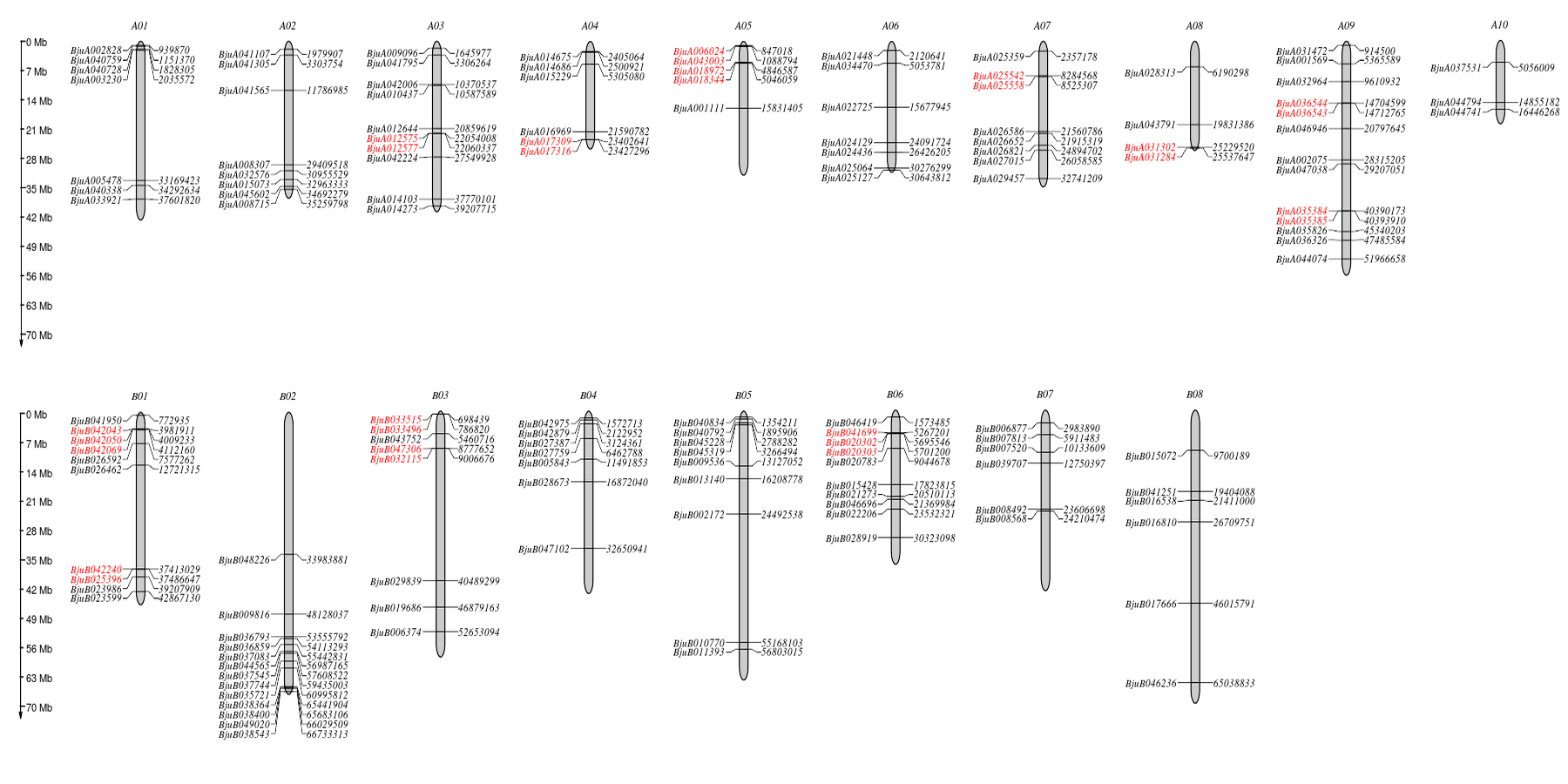

Supplement: Supplemental Information 8 — The physical locations of BjubZIP genes are mapped onto 18 chromosomes (A01–A10, B01–B 08 ). Gene IDs are shown on the side of each chromosome, with positions (Mb) indicated on the left. Genes highlighted in red represent duplicated or tandemly clustered members. [file peerj-14-20518-s008.png]

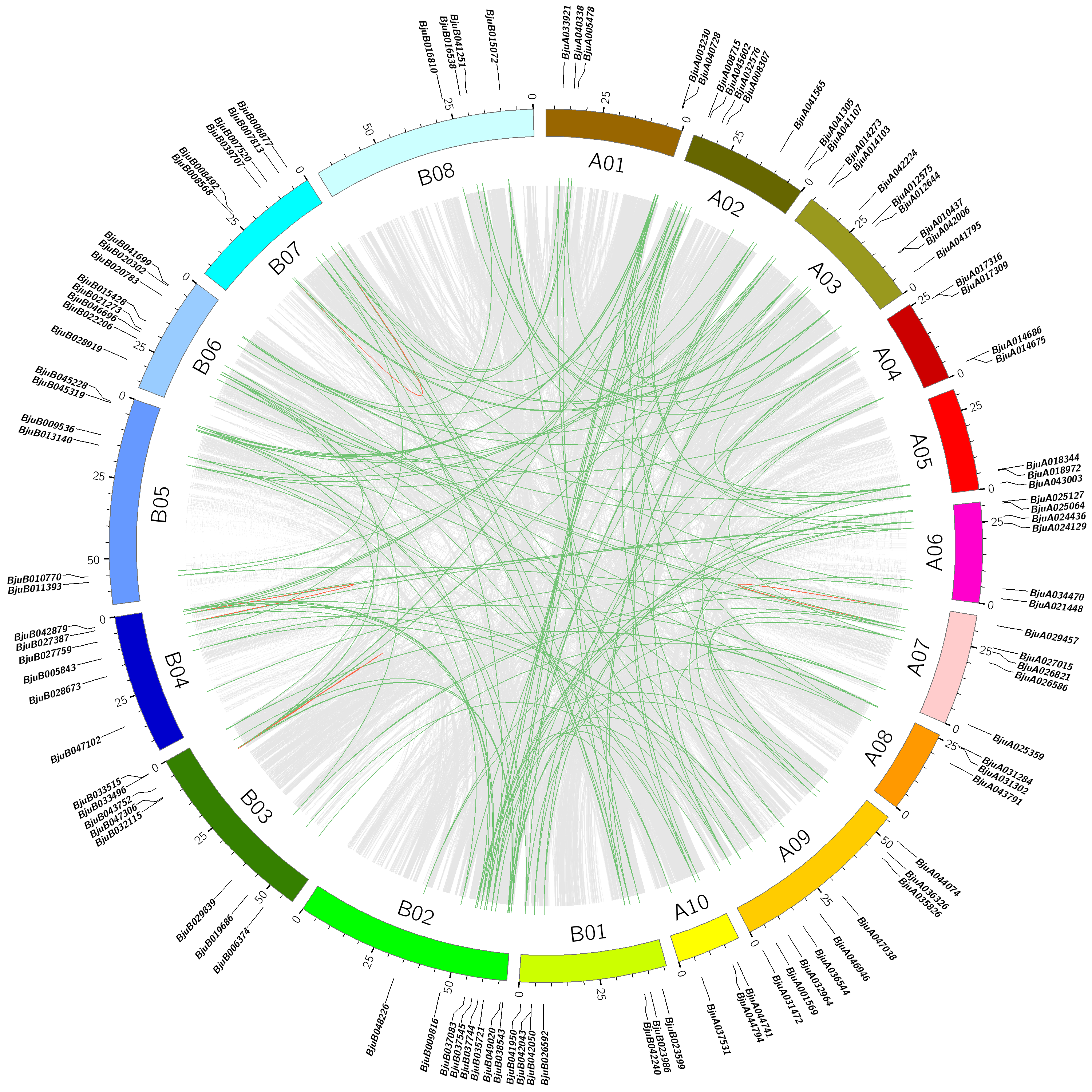

Supplement: Supplemental Information 10 — The gray lines indicate all synteny blocks in the tumorous stem mustard genome, and the green lines indicate duplicated bZIP gene pairs. The chromosome number is indicated at the inside of each chromosome. [file peerj-14-20518-s010.png]

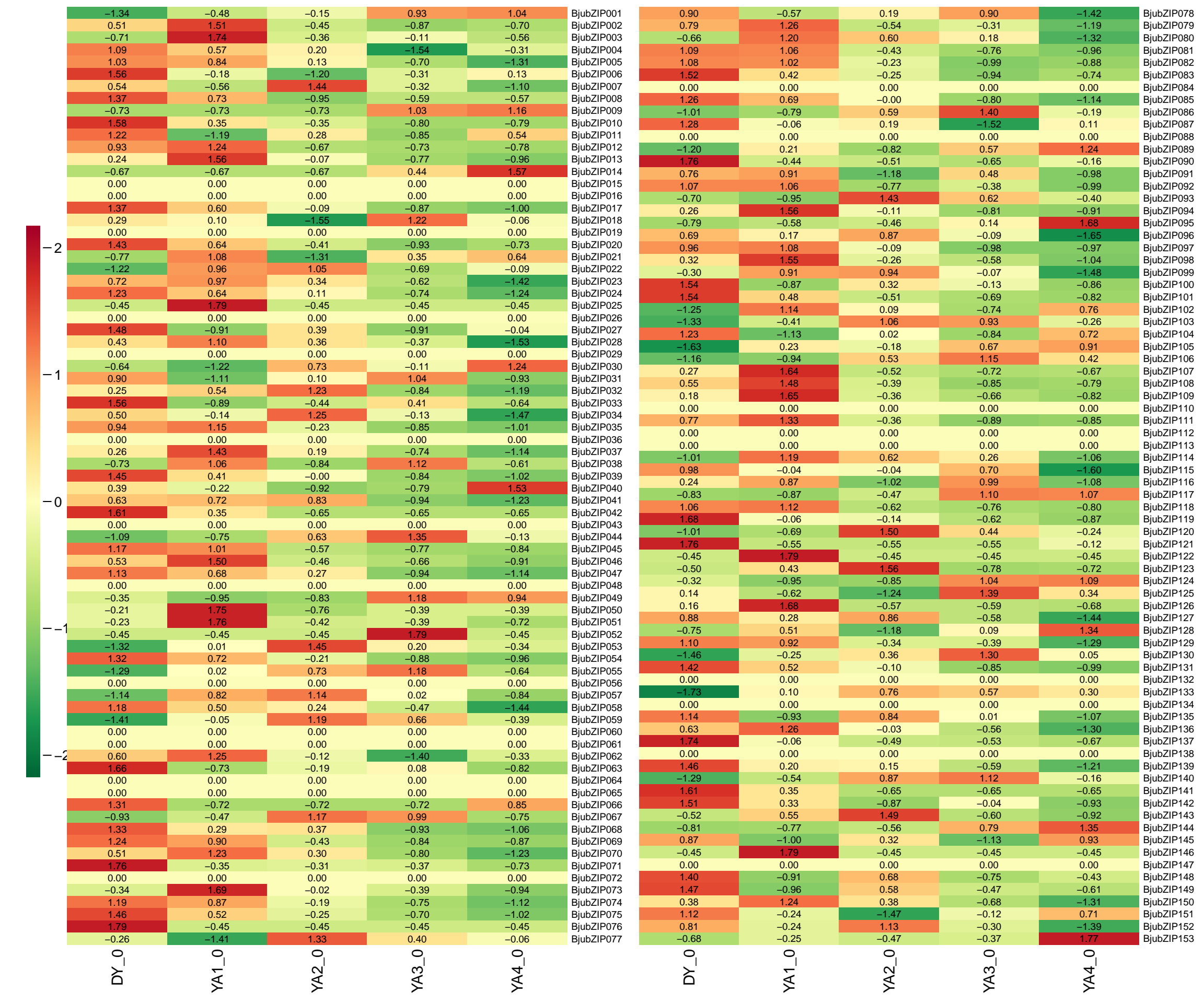

Supplement: Supplemental Information 11 — Heatmap of standardized expression levels (Z-scores) of BjubZIP genes across five developmental stages (DY_0, YA1_0, YA2_0, YA3_0, YA4_0). Red indicates high expression, green indicates low expression, and yellow indicates intermediate expression. [file peerj-14-20518-s011.pdf]

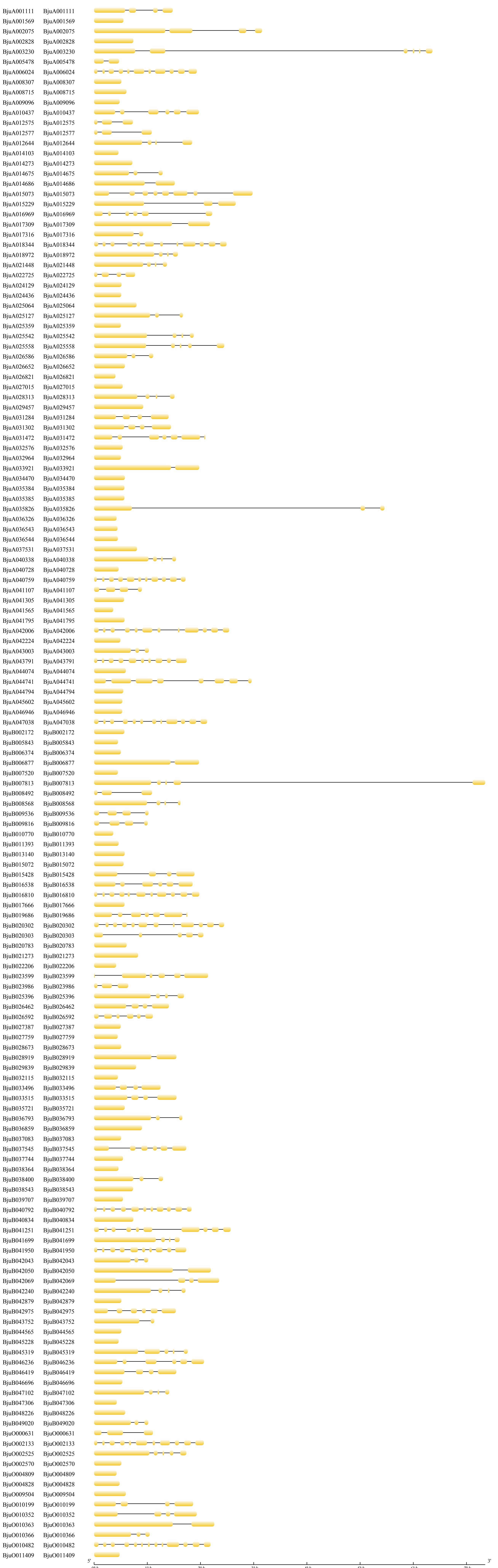

Supplement: Supplemental Information 17 [file peerj-14-20518-s017.zip › bzip raw file/exon/YKUMRZ.pdf]

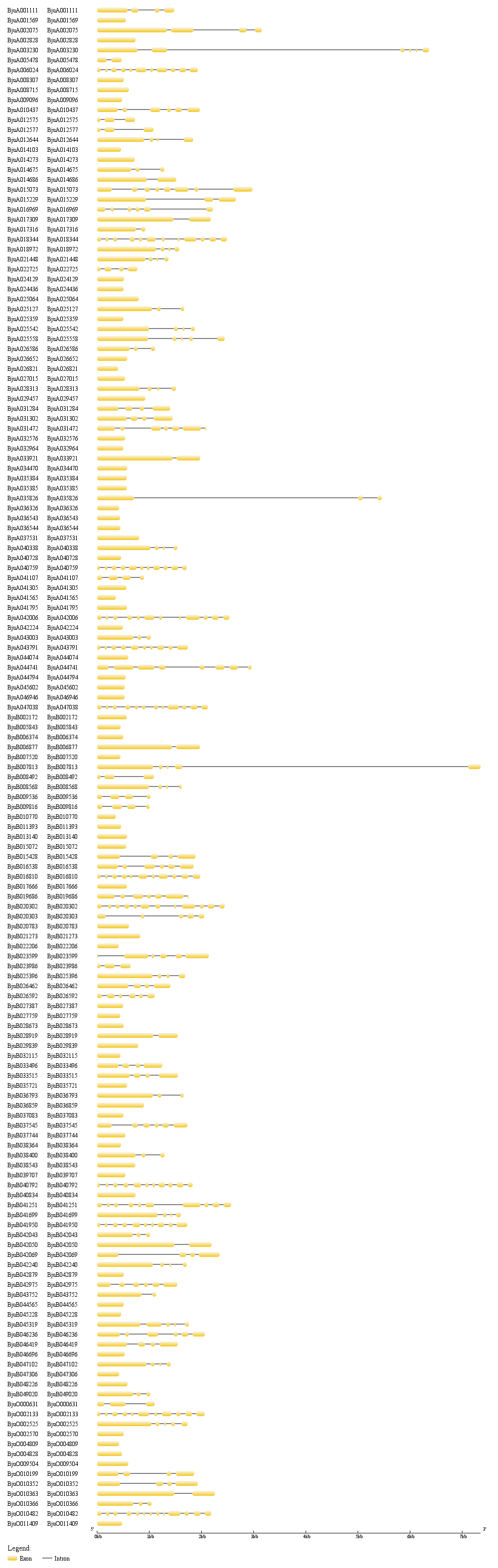

Supplement: Supplemental Information 17 [file peerj-14-20518-s017.zip › bzip raw file/exon/YKUMRZ.png]

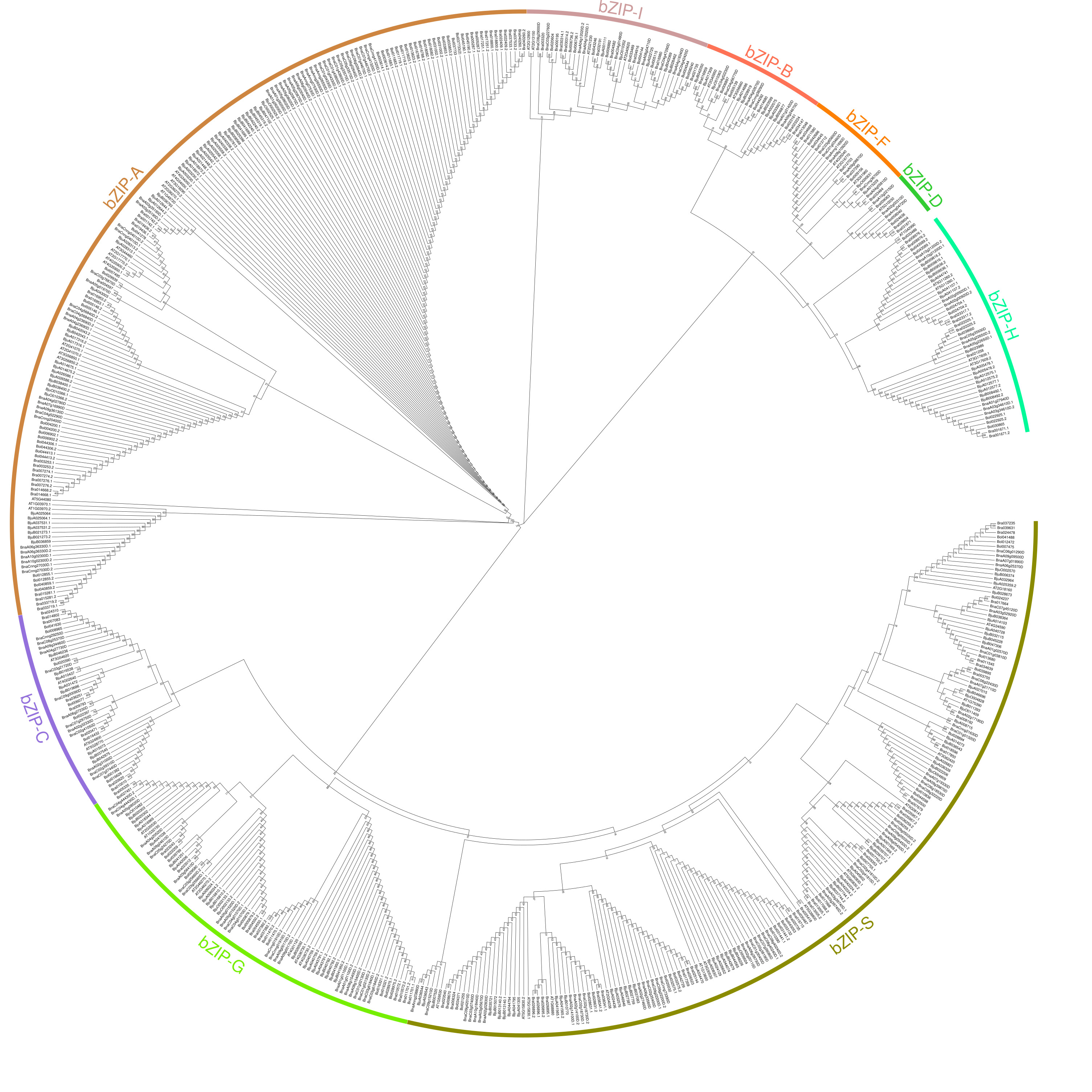

Supplement: Supplemental Information 17 [file peerj-14-20518-s017.zip › bzip raw file/exon/bZIP.jpg]

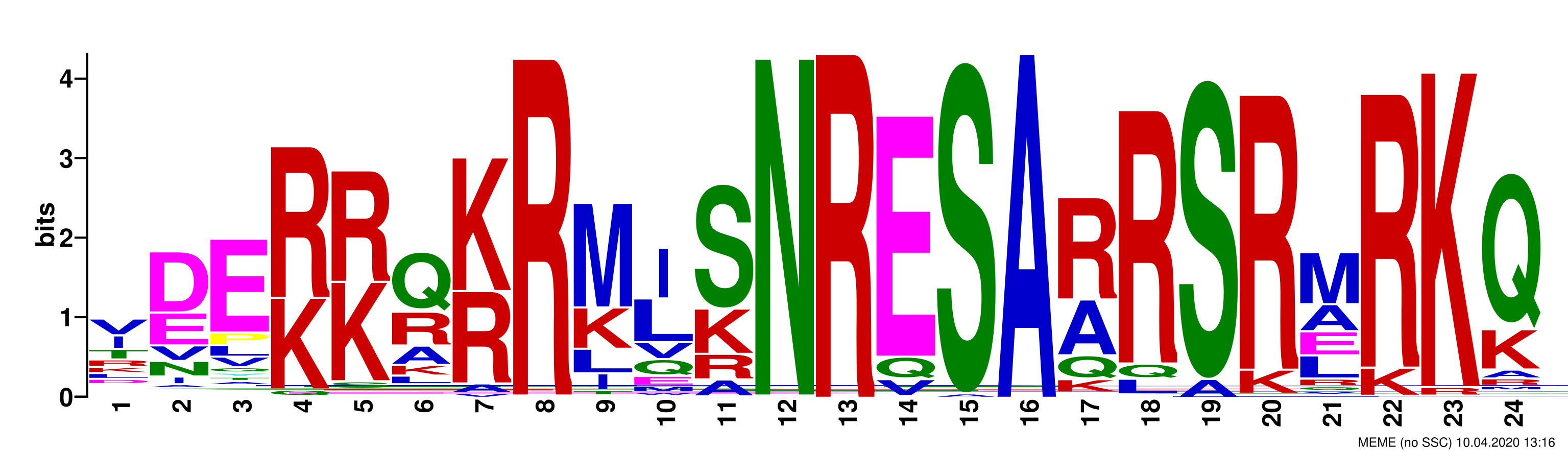

Supplement: Supplemental Information 17 [file peerj-14-20518-s017.zip › bzip raw file/motif/JPG/1.jpg]

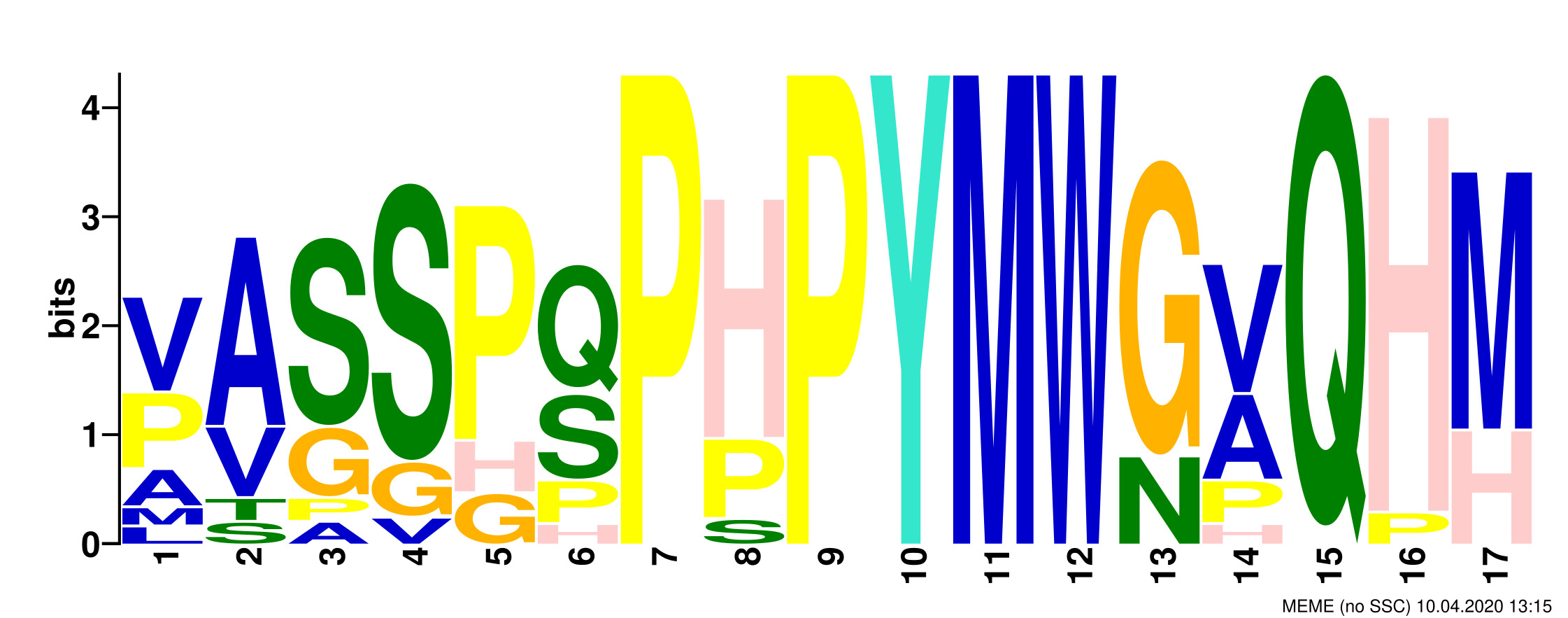

Supplement: Supplemental Information 17 [file peerj-14-20518-s017.zip › bzip raw file/motif/JPG/10.jpg]

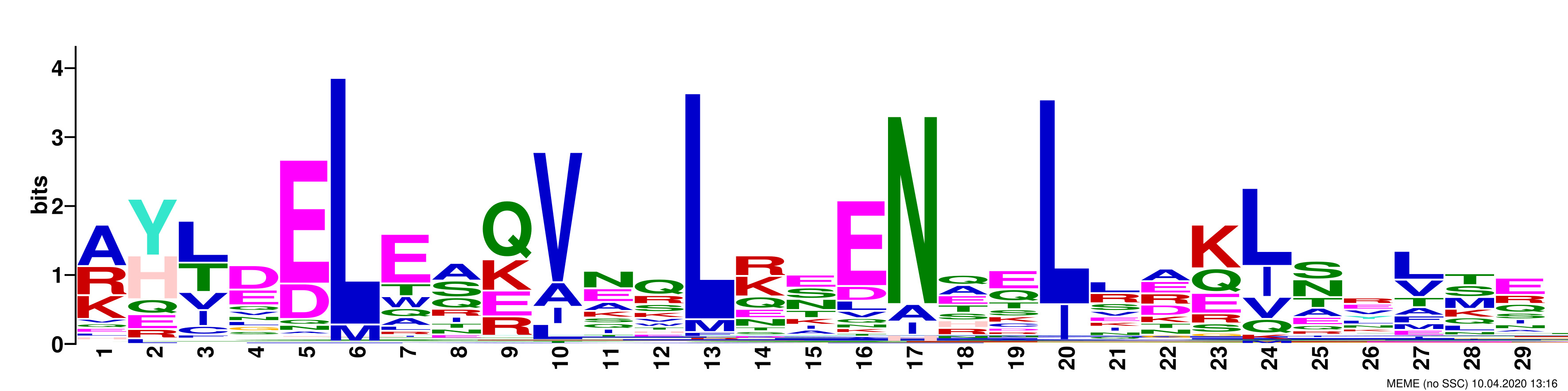

Supplement: Supplemental Information 17 [file peerj-14-20518-s017.zip › bzip raw file/motif/JPG/2.jpg]

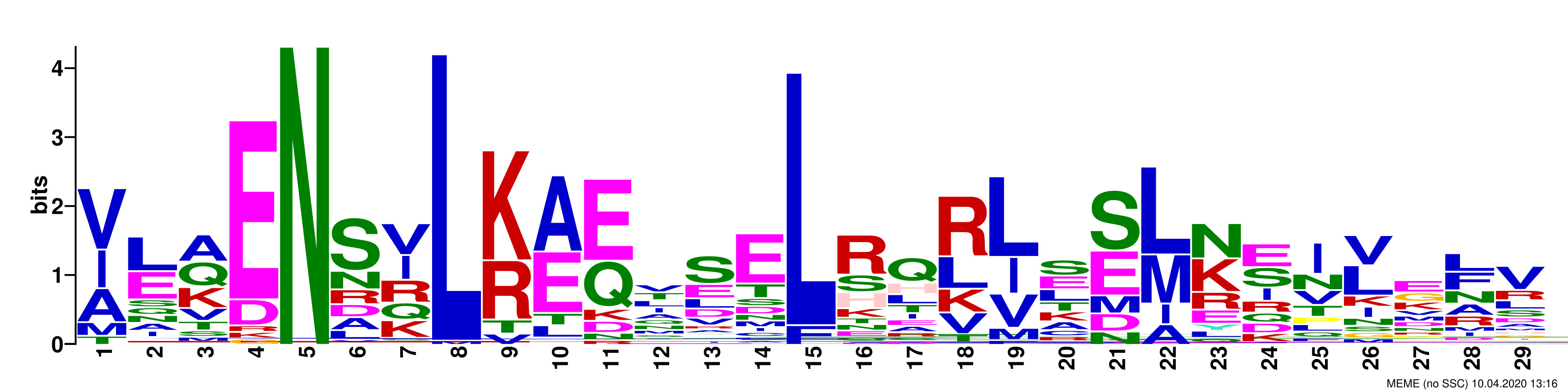

Supplement: Supplemental Information 17 [file peerj-14-20518-s017.zip › bzip raw file/motif/JPG/3.jpg]

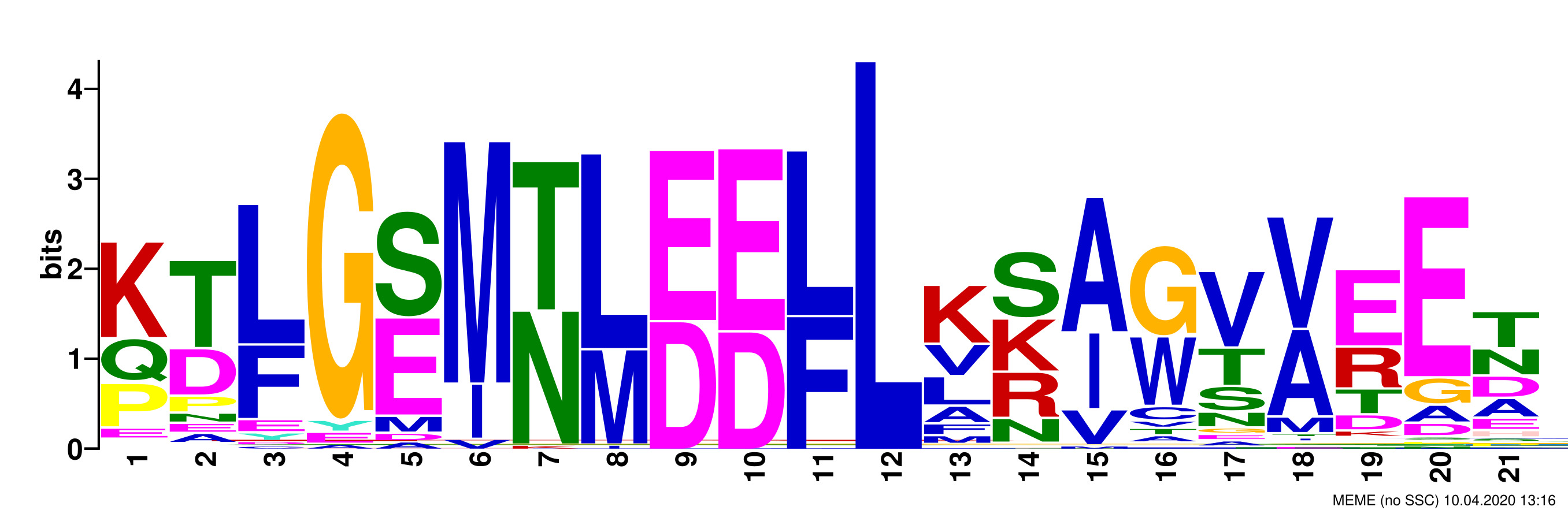

Supplement: Supplemental Information 17 [file peerj-14-20518-s017.zip › bzip raw file/motif/JPG/4.jpg]

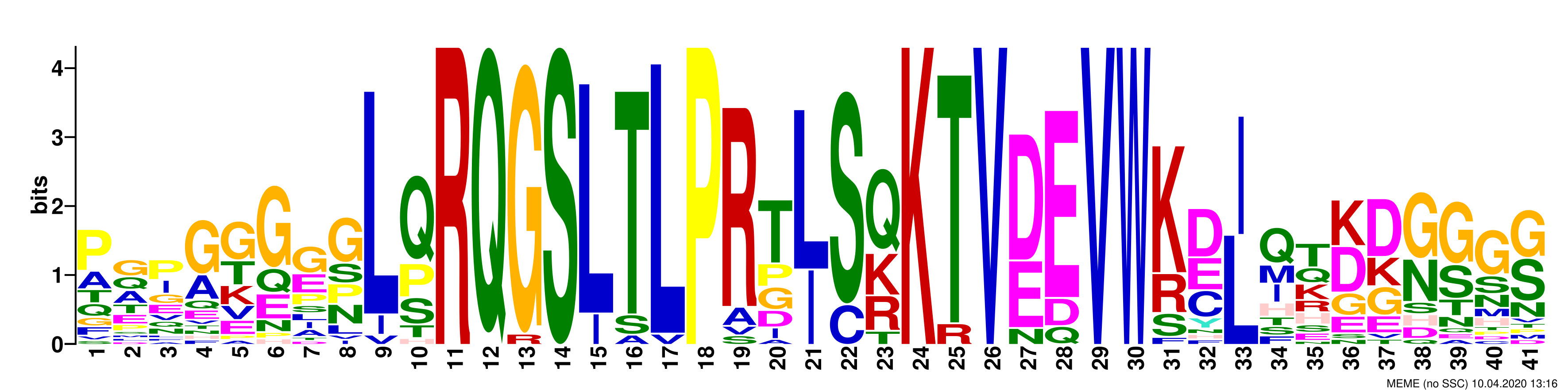

Supplement: Supplemental Information 17 [file peerj-14-20518-s017.zip › bzip raw file/motif/JPG/5.jpg]

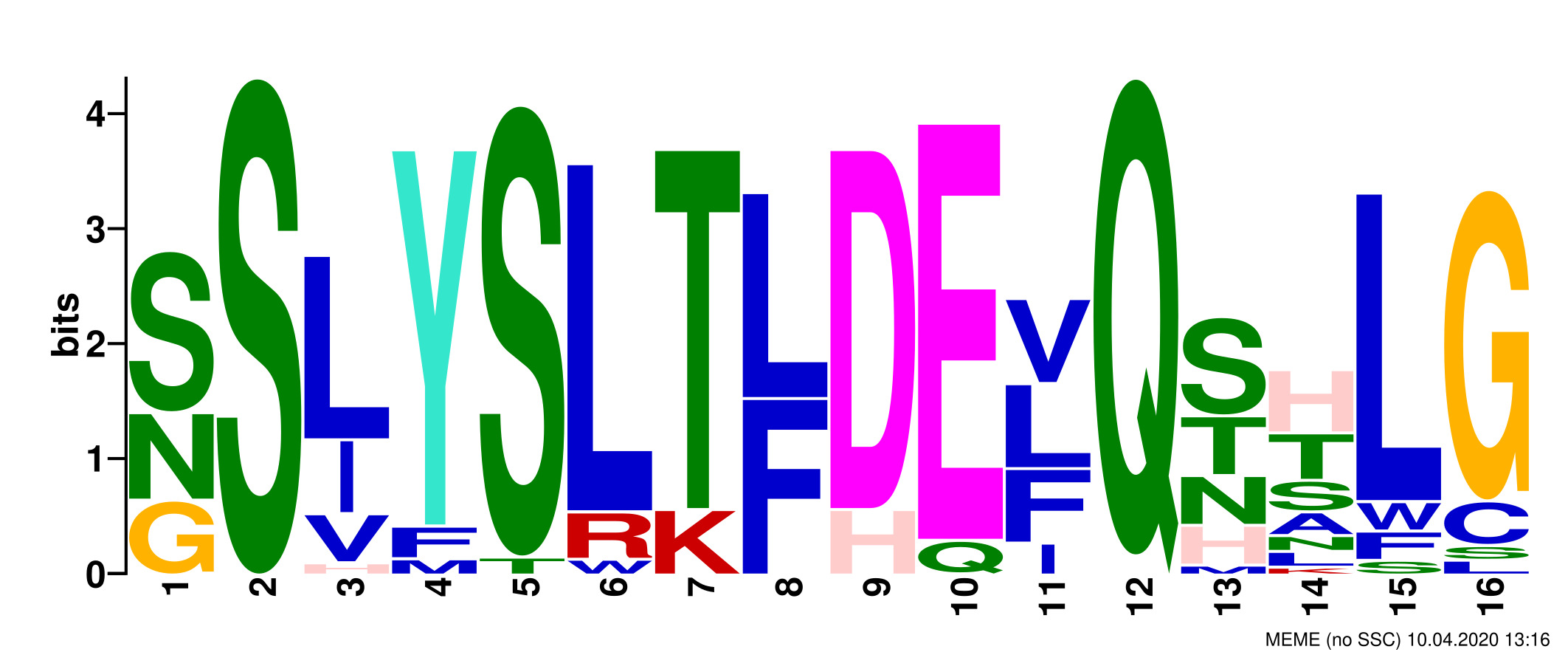

Supplement: Supplemental Information 17 [file peerj-14-20518-s017.zip › bzip raw file/motif/JPG/6.jpg]

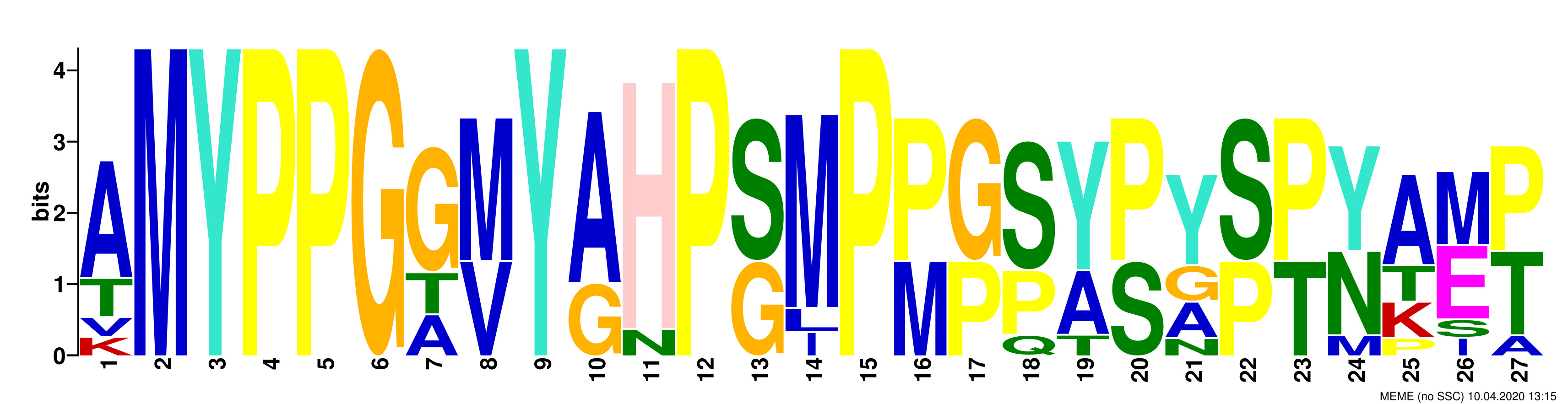

Supplement: Supplemental Information 17 [file peerj-14-20518-s017.zip › bzip raw file/motif/JPG/7.jpg]

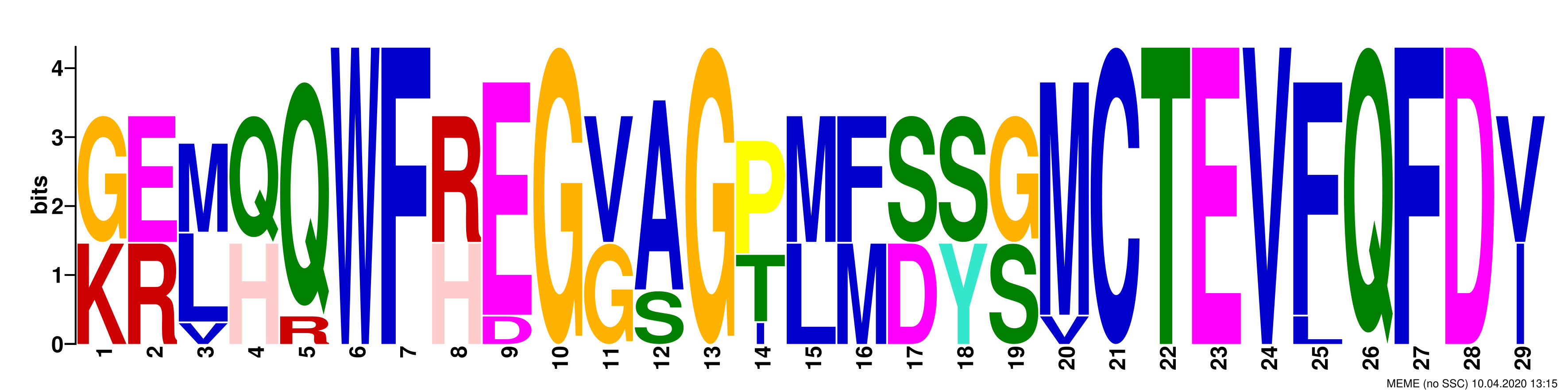

Supplement: Supplemental Information 17 [file peerj-14-20518-s017.zip › bzip raw file/motif/JPG/8.jpg]

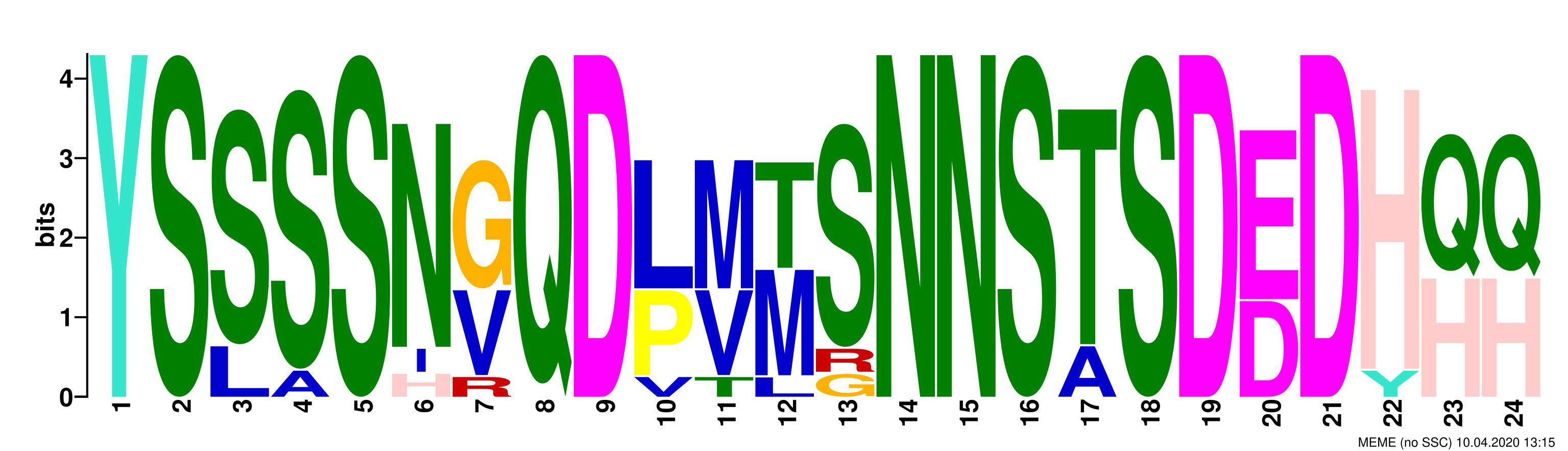

Supplement: Supplemental Information 17 [file peerj-14-20518-s017.zip › bzip raw file/motif/JPG/9.jpg]

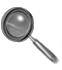

Supplement: Supplemental Information 17 [file peerj-14-20518-s017.zip › bzip raw file/motif/Job Status - MEME Suite_files/meme_icon.png]

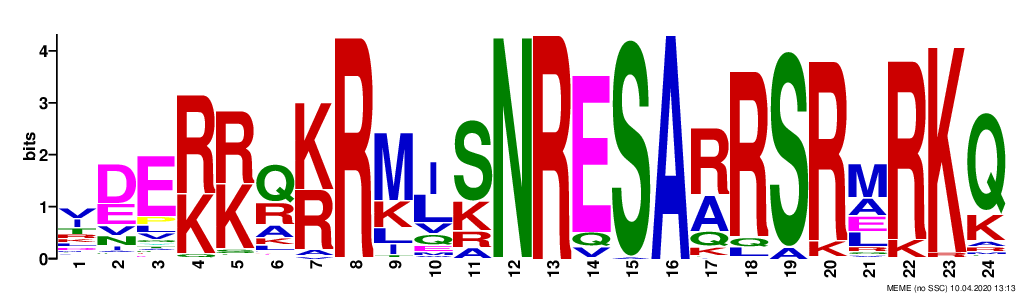

Supplement: Supplemental Information 17 [file peerj-14-20518-s017.zip › bzip raw file/motif/PNG/1.png]

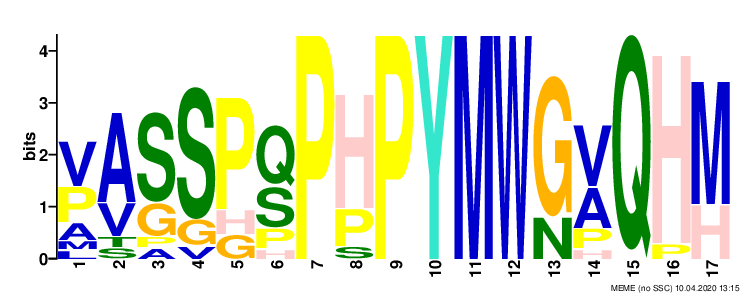

Supplement: Supplemental Information 17 [file peerj-14-20518-s017.zip › bzip raw file/motif/PNG/10.png]

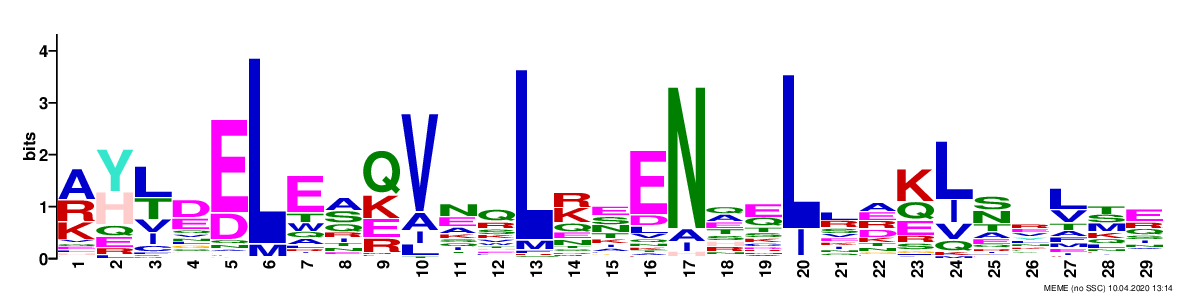

Supplement: Supplemental Information 17 [file peerj-14-20518-s017.zip › bzip raw file/motif/PNG/2.png]

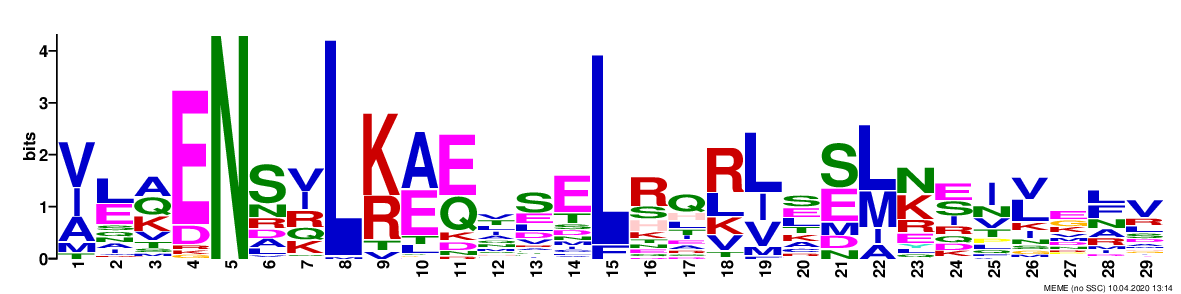

Supplement: Supplemental Information 17 [file peerj-14-20518-s017.zip › bzip raw file/motif/PNG/3.png]

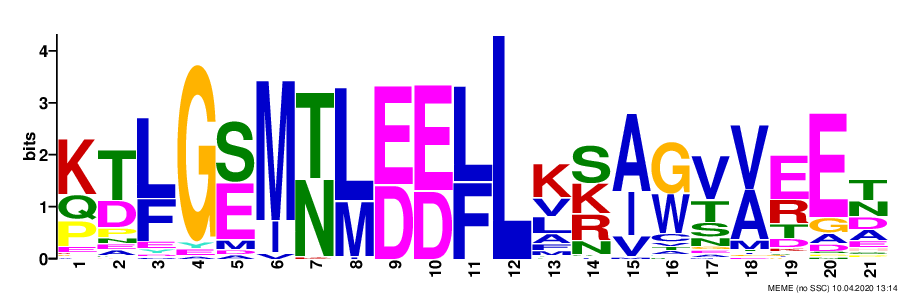

Supplement: Supplemental Information 17 [file peerj-14-20518-s017.zip › bzip raw file/motif/PNG/4.png]

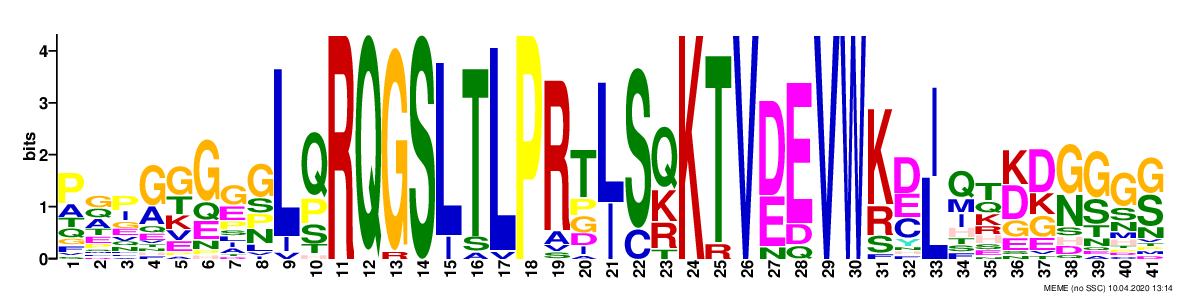

Supplement: Supplemental Information 17 [file peerj-14-20518-s017.zip › bzip raw file/motif/PNG/5.png]

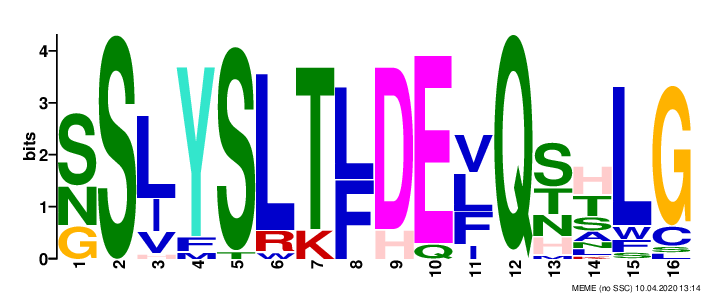

Supplement: Supplemental Information 17 [file peerj-14-20518-s017.zip › bzip raw file/motif/PNG/6.png]

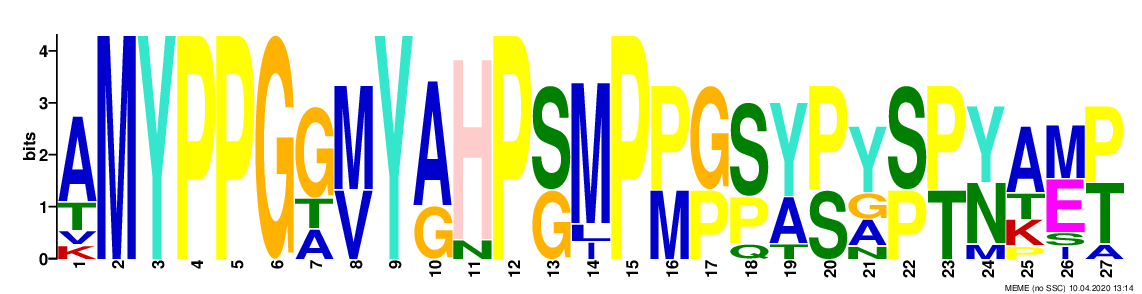

Supplement: Supplemental Information 17 [file peerj-14-20518-s017.zip › bzip raw file/motif/PNG/7.png]

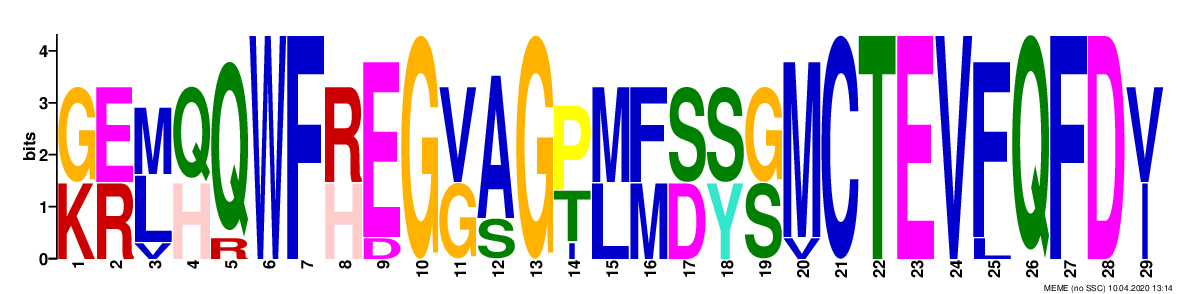

Supplement: Supplemental Information 17 [file peerj-14-20518-s017.zip › bzip raw file/motif/PNG/8.png]

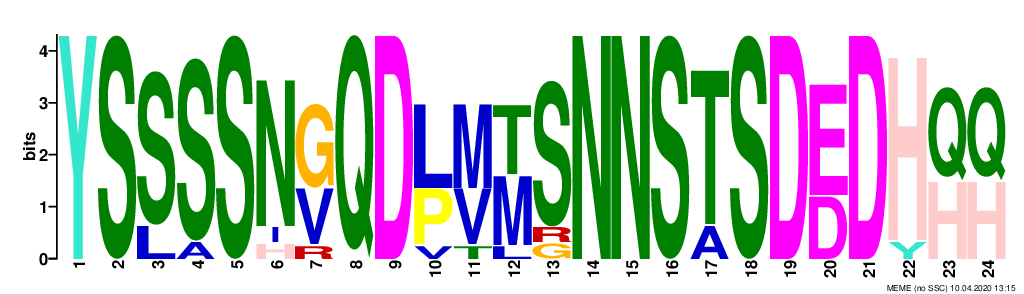

Supplement: Supplemental Information 17 [file peerj-14-20518-s017.zip › bzip raw file/motif/PNG/9.png]

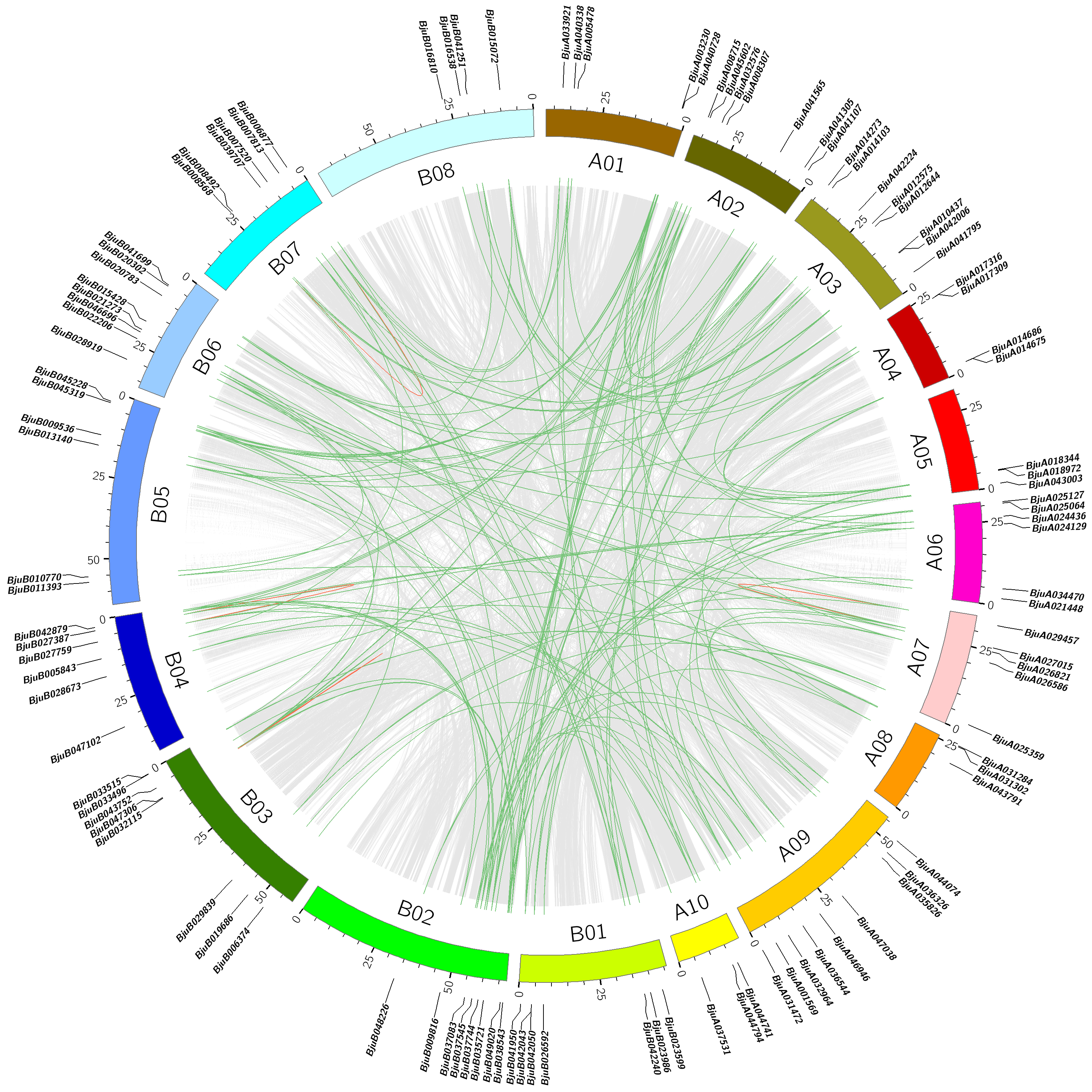

Supplement: Supplemental Information 18 [file peerj-14-20518-s018.zip › circos/bZIP_circos.png]

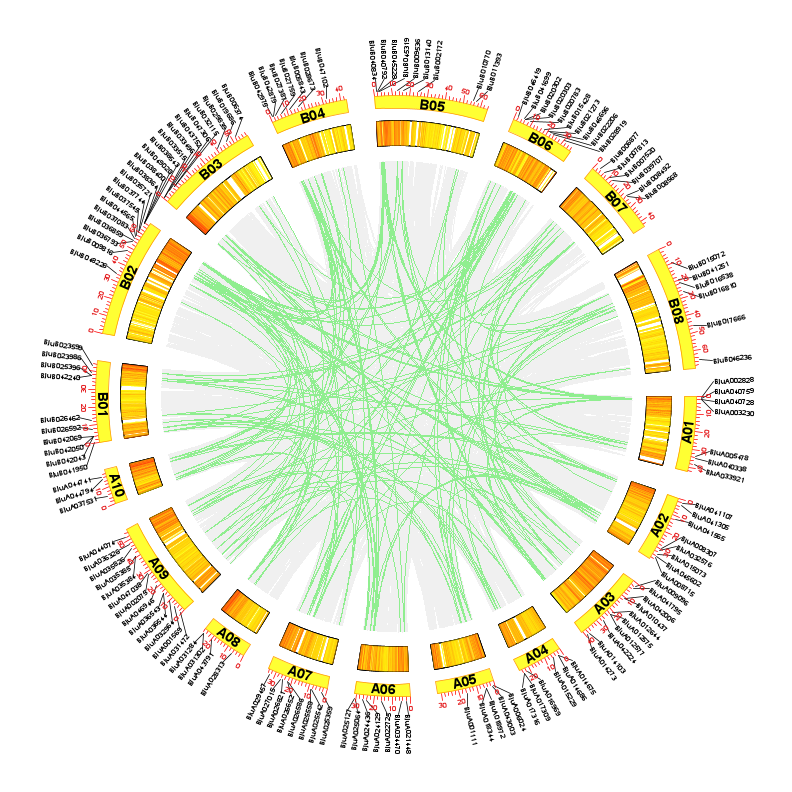

Supplement: Supplemental Information 18 [file peerj-14-20518-s018.zip › circos/circos_photograph/1(1).png]

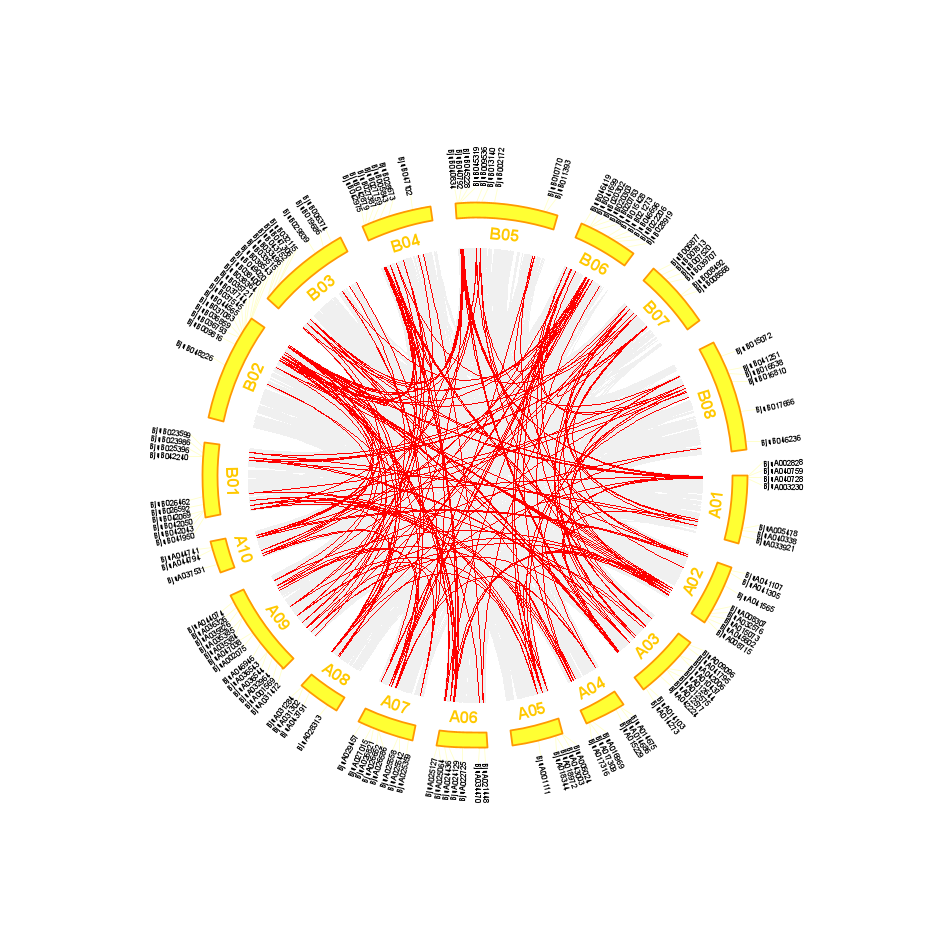

Supplement: Supplemental Information 18 [file peerj-14-20518-s018.zip › circos/circos_photograph/1.png]
