# Supplementary material for: Genome-wide systematic characterization of bZIP transcription factors and their expression profiles during stem in tumorous stem mustard
Source: PeerJ. 2026 Jan 14;14:e20518. doi: 10.7717/peerj.20518 (PMC12811965; doi:10.7717/peerj.20518)
Supplement: Supplemental Information 9 — Cis-acting regulatory elements identified within 1500 bp upstream promoter regions of BjubZIP genes in tumorous stem mustard. Different colors represent distinct types of cis-elements. [file peerj-14-20518-s009.pdf]

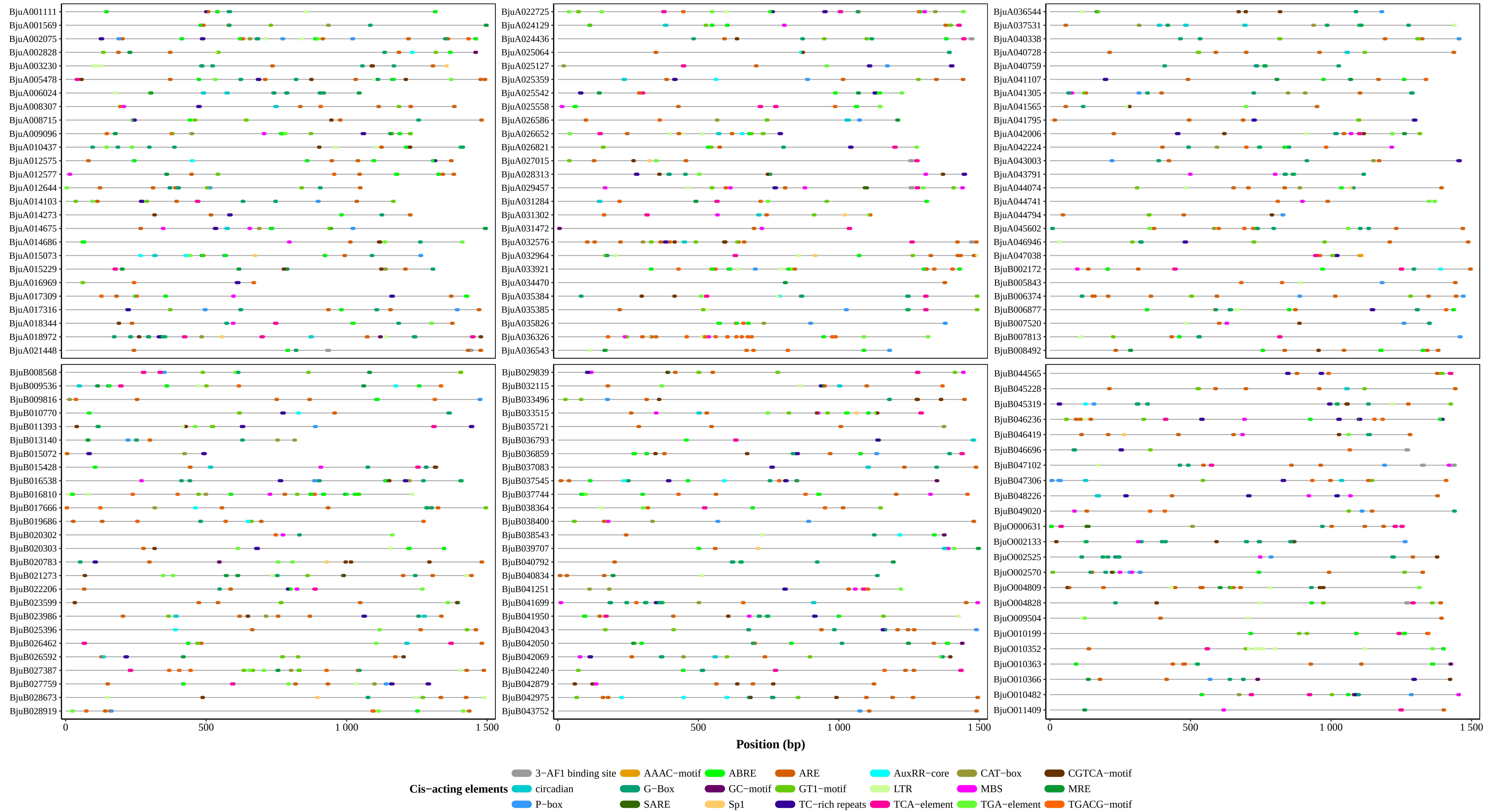
BjuA001111
BjuA001569
BjuA002075
BjuA002828
BjuA003230
BjuA005478
BjuA006024
BjuA008307
BjuA008715
BjuA009096
BjuA010437
BjuA012575
BjuA012577
BjuA012644
BjuA014103
BjuA014273
BjuA014675
BjuA014686
BjuA015073
BjuA015229
BjuA016969
BjuA017309
BjuA017316
BjuA018344
BjuA018972
BjuA021448
BjuA022725
BjuA024129
BjuA024436
BjuA025064
BjuA025127
BjuA025359
BjuA025542
BjuA025558
BjuA026586
BjuA026652
BjuA026821
BjuA027015
BjuA028313
BjuA029457
BjuA031284
BjuA031302
BjuA031472
BjuA032576
BjuA032964
BjuA033921
BjuA034470
BjuA035384
BjuA035385
BjuA035826
BjuA036326
BjuA036543
BjuA036544
BjuA037531
BjuA040338
BjuA040728
BjuA040759
BjuA041107
BjuA041305
BjuA041565
BjuA041795
BjuA042006
BjuA042224
BjuA043003
BjuA043791
BjuA044074
BjuA044741
BjuA044794
BjuA045602
BjuA046946
BjuA047038
BjuB002172
BjuB005843
BjuB006374
BjuB006877
BjuB007520
BjuB007813
BjuB008492
BjuB008568
BjuB009536
BjuB009816
BjuB010770
BjuB011393
BjuB013140
BjuB015072
BjuB015428
BjuB016538
BjuB016810
BjuB017666
BjuB019686
BjuB020302
BjuB020303
BjuB020783
BjuB021273
BjuB022206
BjuB023599
BjuB023986
BjuB025396
BjuB026462
BjuB026592
BjuB027387
BjuB027759
BjuB028673
BjuB028919
BjuB029839
BjuB032115
BjuB033496
BjuB033515
BjuB035721
BjuB036793
BjuB036859
BjuB037083
BjuB037545
BjuB037744
BjuB038364
BjuB038400
BjuB038543
BjuB039707
BjuB040792
BjuB040834
BjuB041251
BjuB041699
BjuB041950
BjuB042043
BjuB042050
BjuB042069
BjuB042240
BjuB042879
BjuB042975
BjuB043752
BjuB044565
BjuB045228
BjuB045319
BjuB046236
BjuB046419
BjuB046696
BjuB047102
BjuB047306
BjuB048226
BjuB049020
BjuO000631
BjuO002133
BjuO002525
BjuO002570
BjuO004809
BjuO004828
BjuO009504
BjuO010199
BjuO010352
BjuO010363
BjuO010366
BjuO010482
BjuO011409
0
500
1 000
1 500
Position (bp)
Cis−acting elements
3−AF1 binding site
AAAC−motif
ABRE
ARE
AuxRR−core
CAT−box
CGTCA−motif
circadian
G−Box
GC−motif
GT1−motif
LTR
MBS
MRE
P−box
SARE
Sp1
TC−rich repeats
TCA−element
TGA−element
TGACG−motif
