# Supplementary material for: Genome-wide systematic characterization of bZIP transcription factors and their expression profiles during stem in tumorous stem mustard
Source: PeerJ. 2026 Jan 14;14:e20518. doi: 10.7717/peerj.20518 (PMC12811965; doi:10.7717/peerj.20518)
Supplement: Supplemental Information 17 [file peerj-14-20518-s017.zip › bzip raw file/Motif.docx]

| Motif1 | - 5.3e-2196 - 151 sites | 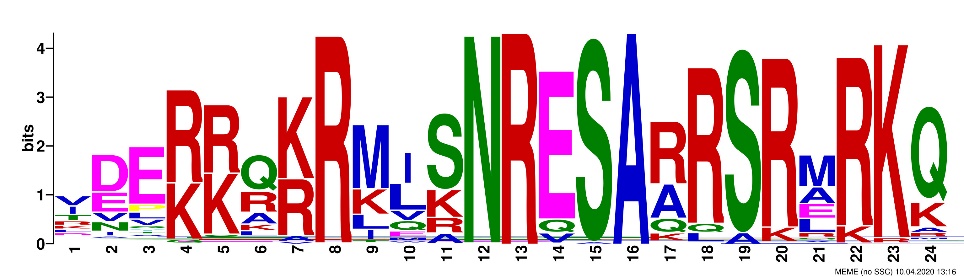 |
| --- | --- | --- |
| Motif2 | - 2.2e-1197 - 157 sites | 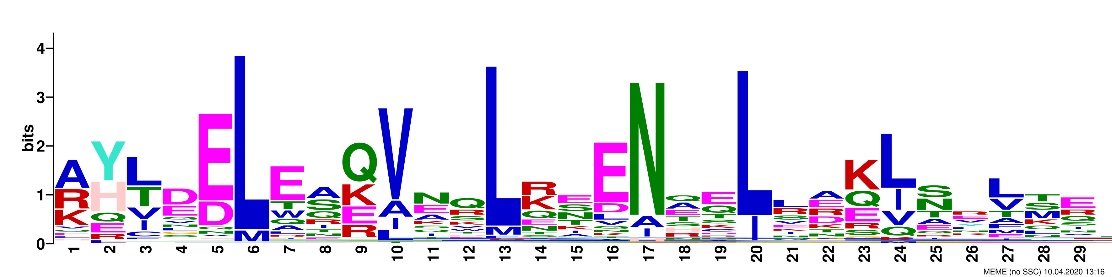 |
| Motif3 | - 1.6e-522 - 69 sites | 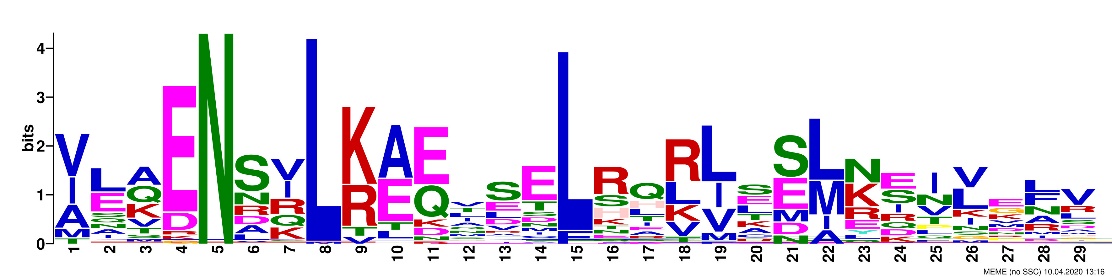 |
| Motif4 | - 8.9e-516 - 56 sites | 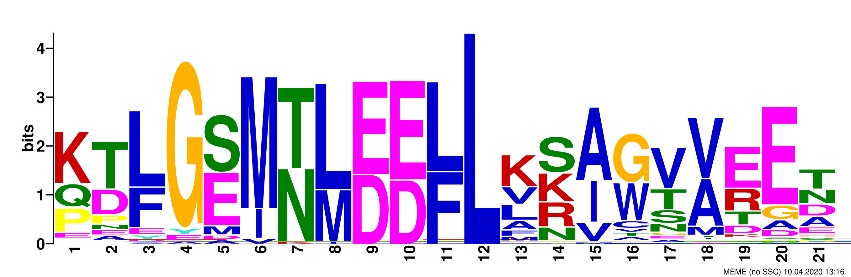 |
| Motif5 | - 5.4e-446 - 25 sites | 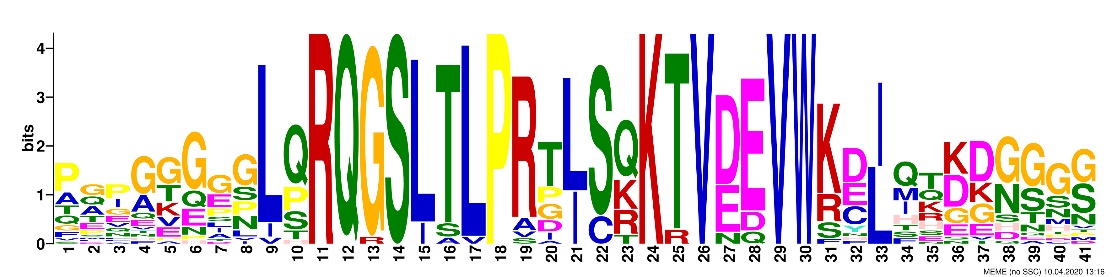 |
| Motif6 | - 6.4e-181 - 26 sites | 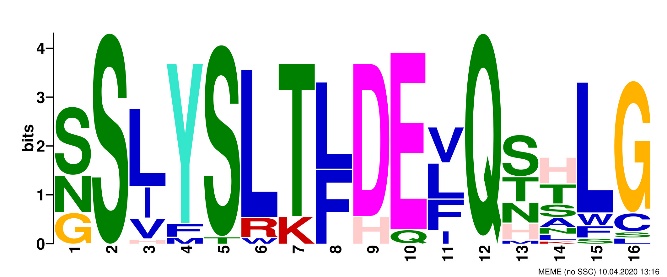 |
| Motif7 | - 1.3e-119 - 10 sites | 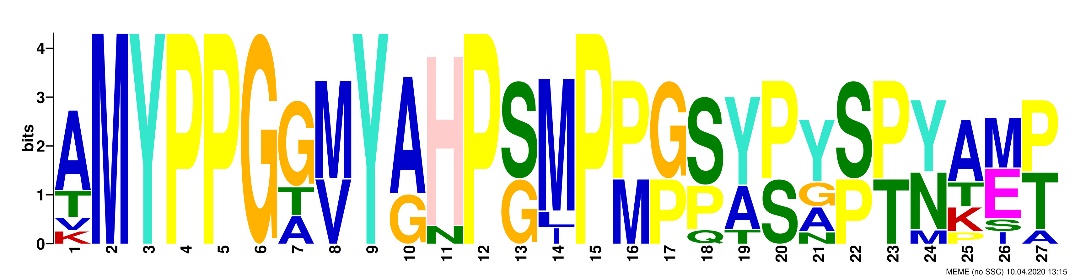 |
| Motif8 | - 2.6e-148 - 9 sites | 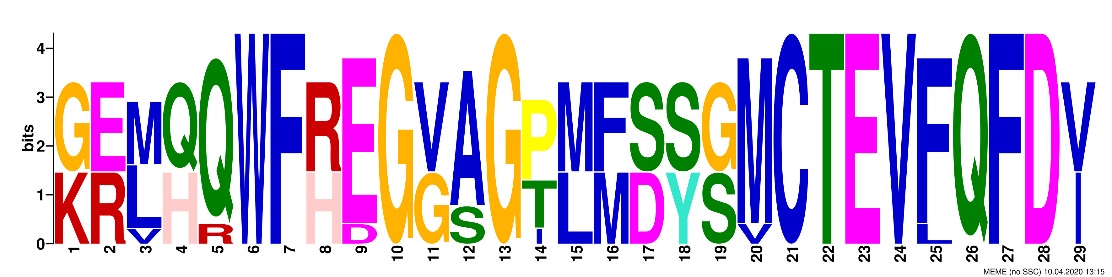 |
| Motif9 | - 8.8e-121 - 11 sites | 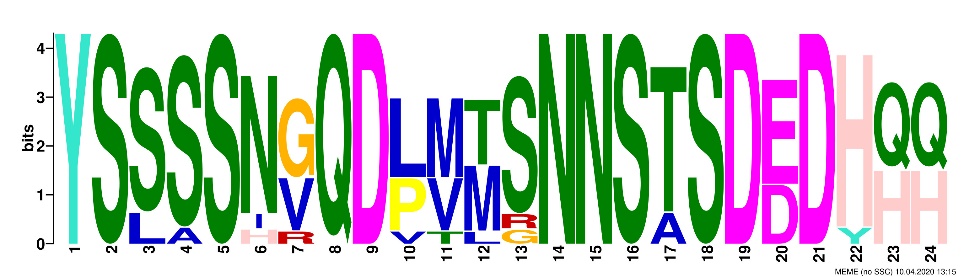 |
| Motif10 | - 3.4e-112 - 13 sites | 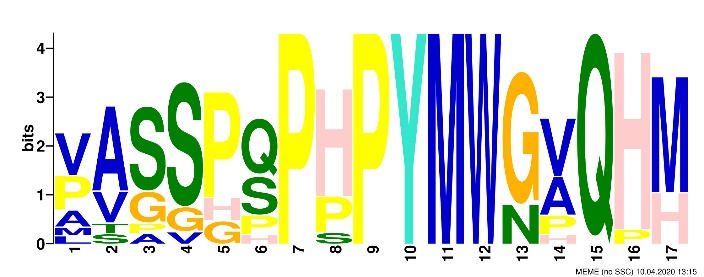 |
