# Supplementary material for: Genome-wide systematic characterization of bZIP transcription factors and their expression profiles during stem in tumorous stem mustard
Source: PeerJ. 2026 Jan 14;14:e20518. doi: 10.7717/peerj.20518 (PMC12811965; doi:10.7717/peerj.20518)
Supplement: Supplemental Information 17 [file peerj-14-20518-s017.zip › bzip raw file/motif/Job Status - MEME Suite.html]

Job Status - MEME Suite


MEME Suite 5.1.1

▶

▼

Motif Discovery

MEMEDREMEMEME-ChIPGLAM2MoMo

▶

▼

Motif Enrichment

CentriMoAMESpaMoGOMo

▶

▼

Motif Scanning

FIMOMASTMCASTGLAM2Scan

▶

▼

Motif Comparison

Tomtom

▶

▼

Gene Regulation

T-Gene

▶

▼

Manual

OVERVIEW

Motif Discovery

MEMEDREMEMEME-ChIPGLAM2MoMo

Motif Enrichment

CentriMoAMESpaMoGOMo

Motif Scanning

FIMOMASTMCASTGLAM2Scan

Motif Comparison

Tomtom

Gene Regulation

T-Gene

▶

▼

Guides & Tutorials

AMECentriMoT-GeneDREMEFIMOGLAM2GT-ScanMEME-ChIP

▶

▼

Sample Outputs

Motif Discovery

MEME SampleDREME SampleMEME-ChIP SampleGLAM2 SampleMoMo Sample

Motif Enrichment

CentriMo SampleAME SampleSpaMo SampleGOMo Sample

Motif Scanning

FIMO SampleMAST SampleMCAST SampleGLAM2Scan Sample

Motif Comparison

Tomtom Sample

Gene Regulation

T-Gene Sample

▶

▼

File Format Reference

FASTA SequenceMEME Motif formatAME output formatsCentriMo output formatsFIMO output formatsGOMo output formatsMCAST output formatsMEME-ChIP output formatsMoMo output formatsSpaMo output formatsT-Gene output formatsTomtom output formatsPeptide-Spectrum MatchCustom AlphabetMarkov Background ModelPosition-specific prior (PSP)Dirichlet MixturesOther Supported Formats

▶

▼

Databases

Motif DatabasesSequence DatabasesGOMo Sequence DatabasesT-Gene Genomes and Tissue Panels

▶

▼

Download & Install

Download MEME Suite and DatabasesCopyright NoticeInstallation GuideRelease NotesRelease Announcement GroupCommercial Licensing

▶

▼

Help

Q&A ForumEmail WebmasterEmail Developers

▶

▼

Alternate Servers

Main Server->View Current LoadAlternate Server->View Current LoadGenQuest (France)

▶

▼

Authors & Citing

AuthorsCiting the MEME Suite

▶

▼

Recent Jobs

✕MEME下午4:00

Clear All

↪ Previous version 5.1.0


# Javascript is disabled! ☹

The MEME Suite web application requires the use of JavaScript but
Javascript doesn't seem to be available on your browser.

Please re-enable Javascript to use the MEME Suite.

# MEME

## Multiple Em for Motif Elicitation

The job you requested could not be found. If you
have followed a link in your verification email it is probable that the
job has expired and the output has been deleted to clear up
resources.
Please wait. Your MEME job is now queued
awaiting available resources.
You may bookmark this page or use the **Recent Jobs** menu at the left
to access your job's results.
Please wait. Your MEME job is now running.
Further details may be available below.
You may bookmark this page or use the **Recent Jobs** menu at the left
to access your job's results.
Please wait. Your MEME job has been temporally
suspended and is awaiting resources to continue.
You may bookmark this page or use the **Recent Jobs** menu at the left
to access your job's results.
An error occurred running your MEME job. Further
details may be available below.
Your MEME job is complete. The results should be
displayed below.
The status code received is unknown.

### Job Details

 ...
 ▼

|  |  |
| --- | --- |
| Submitted | 2020/4/10 下午4:00:38 |
| Expires | 2020/4/14 下午4:00:38 |
| Description | bZIP |
| (Primary) Sequences | A set of 153 Protein sequences, between 120 and 680 in length (average length 279.9), from the file bZIP\_pep.fa. |
| Background | A 0-order background model generated from the supplied sequences. |
| Discovery Mode | Classic: optimizes the E-value of the motif information content |
| Site Distribution | Any number of repetitions (of a contributing motif site per sequence) |
| Motif Count | Searching for 10 motifs. |
| Motif Width | Between 6 wide and 50 wide (inclusive). |
