# Supplementary material for: Genome-wide systematic characterization of bZIP transcription factors and their expression profiles during stem in tumorous stem mustard
Source: PeerJ. 2026 Jan 14;14:e20518. doi: 10.7717/peerj.20518 (PMC12811965; doi:10.7717/peerj.20518)
Supplement: Supplemental Information 17 [file peerj-14-20518-s017.zip › bzip raw file/motif/Job Status - MEME Suite_files/index.html]

MEME Job


### Results

- MEME HTML output
- MEME XML output
- MEME text output
- MAST HTML output
- MAST XML output
- MAST text output
- (Primary) Sequences

### Status Messages

### Results

- MEME HTML output
- MEME XML output
- MEME text output
- MAST HTML output
- MAST XML output
- MAST text output
- (Primary) Sequences

### Status Messages

- Parsing arguments
- Arguments ok
- Starting meme  
  `meme bZIP_pep.fa -protein -oc . -nostatus -time 18000 -mod anr -nmotifs 10 -minw 6 -maxw 50 -objfun classic -markov_order 0`
- meme ran successfully in 3052.47 seconds
- Starting mast  
  `mast meme.xml bZIP_pep.fa -oc . -nostatus`
- mast ran successfully in 0.91 seconds
- Done

 
