# Supplementary material for: Genome-wide systematic characterization of bZIP transcription factors and their expression profiles during stem in tumorous stem mustard
Source: PeerJ. 2026 Jan 14;14:e20518. doi: 10.7717/peerj.20518 (PMC12811965; doi:10.7717/peerj.20518)
Supplement: Supplemental Information 17 [file peerj-14-20518-s017.zip › bzip raw file/motif/locations.pdf]

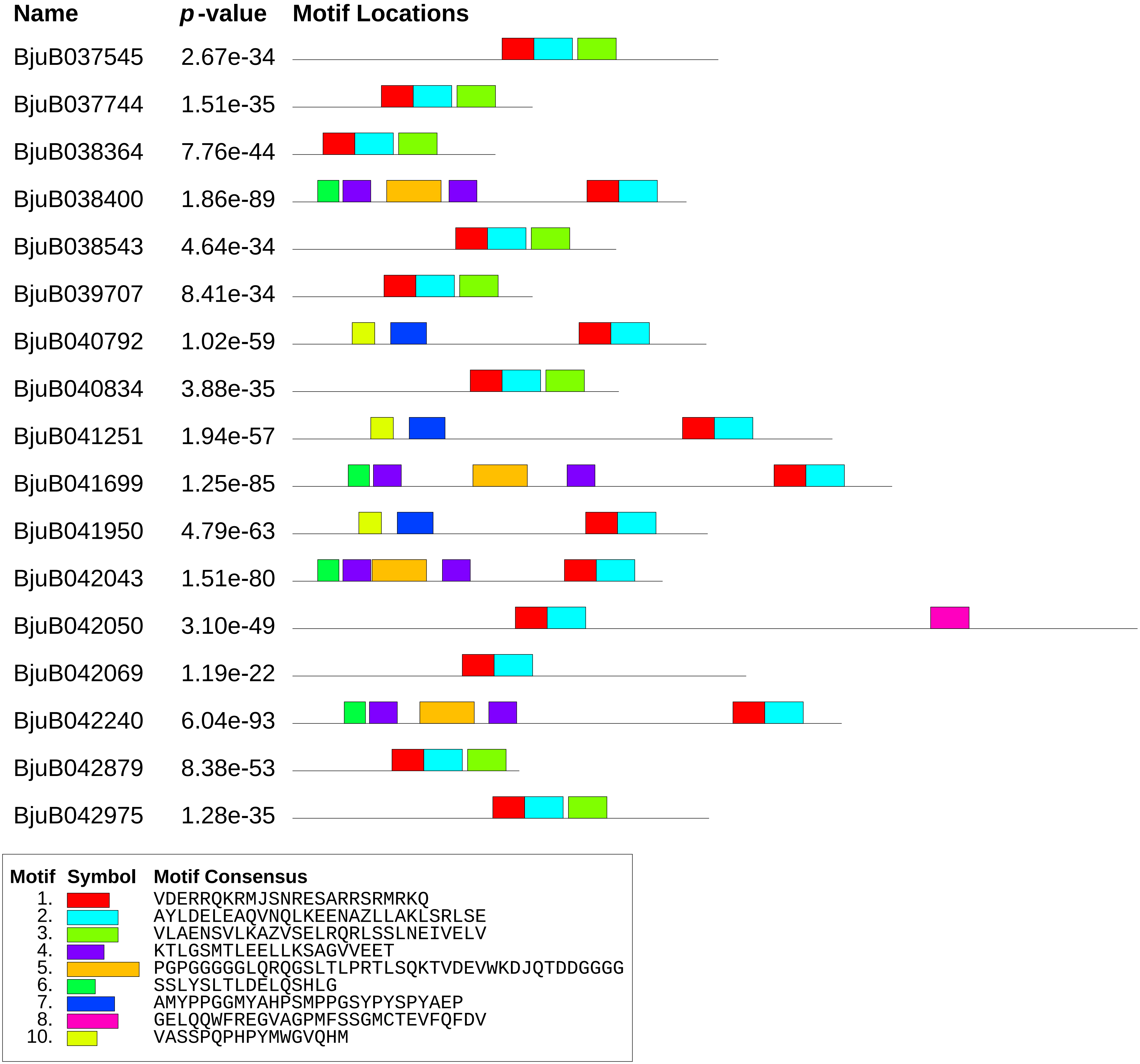
Name
p-value
Motif Locations
BjuB037545 2.67e-34
BjuB037744 1.51e-35
BjuB038364 7.76e-44
BjuB038400 1.86e-89
BjuB038543 4.64e-34
BjuB039707 8.41e-34
BjuB040792 1.02e-59
BjuB040834 3.88e-35
BjuB041251 1.94e-57
BjuB041699 1.25e-85
BjuB041950 4.79e-63
BjuB042043 1.51e-80
BjuB042050 3.10e-49
BjuB042069 1.19e-22
BjuB042240 6.04e-93
BjuB042879 8.38e-53
BjuB042975 1.28e-35
Motif Symbol Motif Consensus
1. VDERRQKRMJSNRESARRSRMRKQ
2. AYLDELEAQVNQLKEENAZLLAKLSRLSE
3. VLAENSVLKAZVSELRQRLSSLNEIVELV
4. KTLGSMTLEELLKSAGVVEET
5. PGPGGGGGLQRQGSLTLPRTLSQKTVDEVWKDJQTDDGGGG
6. SSLYSLTLDELQSHLG
7. AMYPPGGMYAHPSMPPGSYPYSPYAEP
8. GELQQWFREGVAGPMFSSGMCTEVFQFDV
10. VASSPQPHPYMWGVQHM
