# Supplementary material for: Genome-wide systematic characterization of bZIP transcription factors and their expression profiles during stem in tumorous stem mustard
Source: PeerJ. 2026 Jan 14;14:e20518. doi: 10.7717/peerj.20518 (PMC12811965; doi:10.7717/peerj.20518)
Supplement: Supplemental Information 7 — The phylogenetic tree shows the classification of BjubZIP proteins into different subfamilies. Conserved motifs (colored boxes) were identified using MEME, and protein domain annotation highlights the conserved bZIP domains. Gene structures were analyzed with GSDS, where green boxes represent exons and black lines represent introns. [file peerj-14-20518-s007.pdf]

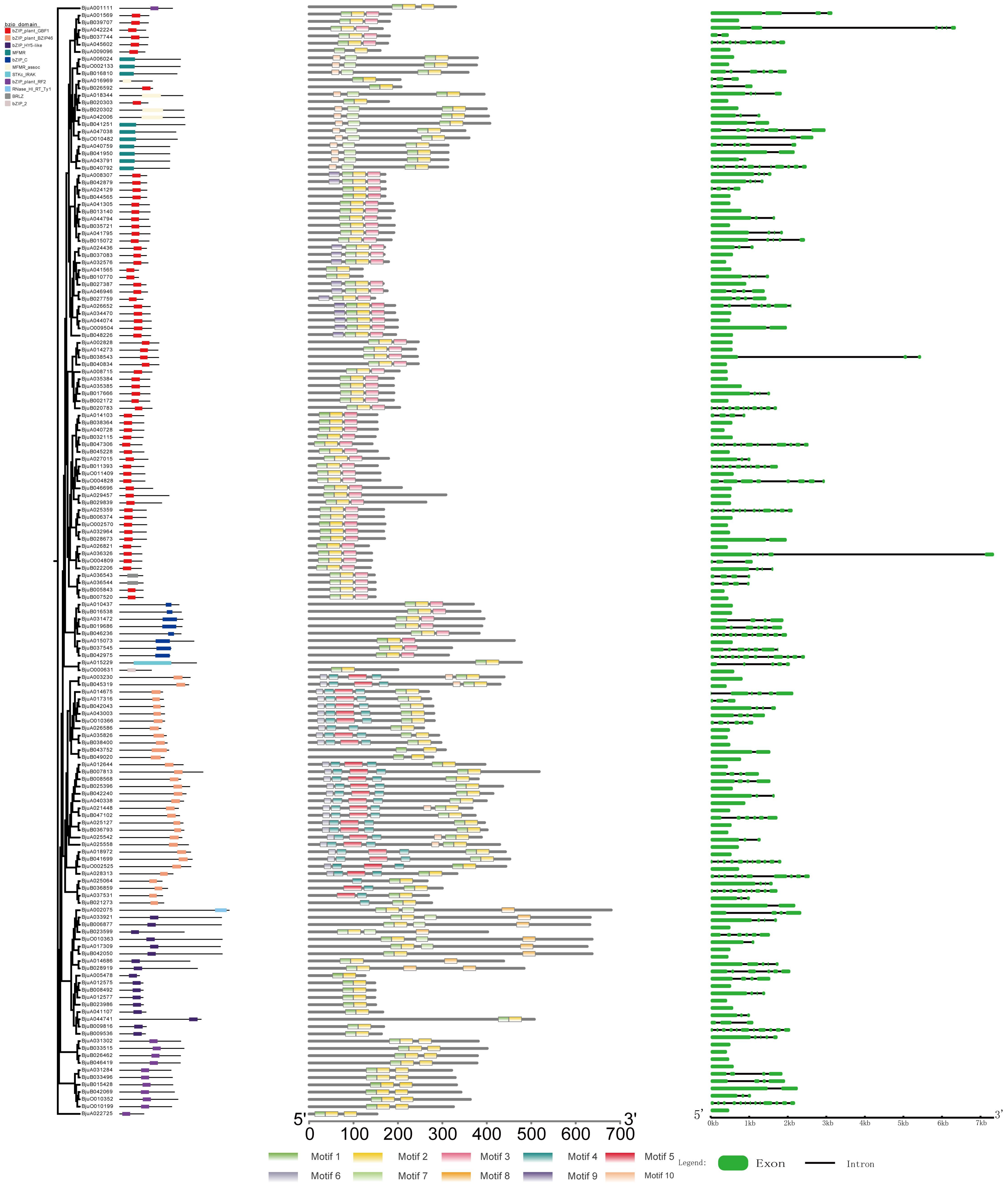
5'
3'
0
100
200
300
400
500
600
700
Motif 1
Motif 2
Motif 3
Motif 4
Motif 5
Motif 6
Motif 7
Motif 8
Motif 9
Motif 10
0kb
1kb
2kb
3kb
4kb
5kb
6kb
7kb
Legend:
Exon
Intron
